# Mammal comparative tendon biology: advances in regulatory mechanisms through a computational modeling

**DOI:** 10.3389/fvets.2023.1175346

**Published:** 2023-04-27

**Authors:** Alessia Peserico, Barbara Barboni, Valentina Russo, Nicola Bernabò, Mohammad El Khatib, Giuseppe Prencipe, Adrián Cerveró-Varona, Arlette Alina Haidar-Montes, Melisa Faydaver, Maria Rita Citeroni, Paolo Berardinelli, Annunziata Mauro

**Affiliations:** Unit of Basic and Applied Sciences, Department of Bioscience and Technology for Food, Agriculture and Environment, University of Teramo, Teramo, Italy

**Keywords:** tendon development, embryonal development, prepubertal development, computational modeling, molecular interaction

## Abstract

There is high clinical demand for the resolution of tendinopathies, which affect mainly adult individuals and animals. Tendon damage resolution during the adult lifetime is not as effective as in earlier stages where complete restoration of tendon structure and property occurs. However, the molecular mechanisms underlying tendon regeneration remain unknown, limiting the development of targeted therapies. The research aim was to draw a comparative map of molecules that control tenogenesis and to exploit systems biology to model their signaling cascades and physiological paths. Using current literature data on molecular interactions in early tendon development, species-specific data collections were created. Then, computational analysis was used to construct Tendon NETworks in which information flow and molecular links were traced, prioritized, and enriched. Species-specific Tendon NETworks generated a data-driven computational framework based on three operative levels and a stage-dependent set of molecules and interactions (embryo–fetal or prepubertal) responsible, respectively, for signaling differentiation and morphogenesis, shaping tendon transcriptional program and downstream modeling of its fibrillogenesis toward a mature tissue. The computational network enrichment unveiled a more complex hierarchical organization of molecule interactions assigning a central role to neuro and endocrine axes which are novel and only partially explored systems for tenogenesis. Overall, this study emphasizes the value of system biology in linking the currently available disjointed molecular data, by establishing the direction and priority of signaling flows. Simultaneously, computational enrichment was critical in revealing new nodes and pathways to watch out for in promoting biomedical advances in tendon healing and developing targeted therapeutic strategies to improve current clinical interventions.

## 1. Introduction

Tendons are fibro-elastic structures that connect muscles to bones allowing movement and conferring resistance even to extreme tensile loads. During the processes of development, the tissue profoundly changes by transforming the tendon from a plastic into a highly specialized structure with a significantly reduced ability to recover homeostasis (regeneration) after injury.

Current therapies for tendinopathies have limited success (1, 2). Most patients do not return to pre-injury activity performance, greatly increasing the likelihood of recurrences as re-ruptures, disability, pain, and impairment of movement. Tendinopathies require prolonged rehabilitation (at least 10 months in humans and up to 18 months in horses), leading to a dramatic socioeconomic impact (3) by combining the absence of a valid therapeutic, rehabilitative, and diagnostic predictive protocol (4) with the rise in life expectancy.

The estimated socioeconomic burden is over €180 billion in the USA and EU, with a forecast of +25% over the next 5 years, because of the absence of an efficacious therapeutic solution and variations in life expectancy, lifestyle, and working stat. In veterinary medicine, 46% of racehorses suffer from tendinopathy and the related reduced performance generates a loss of €400 Bn worldwide (5–7).

The clinical relevance of tendinopathies relies on the inability of adult tissue to activate regenerative processes, which is probably related to its poor cellularity, vascularization, and slow metabolism (4, 8–12).

Indeed, tendon healing predominantly relies on reparative processes during the adult age, which involves the deposition of fibrous disorganized tissue (scar) instead of a hierarchically organized extracellular matrix (ECM). Repairing instead of regenerative healing compromises the biomechanical properties (4, 13–15). On the other hand, tendon healing results to be greatly efficient during the early stage of an individual lifetime. In particular, during the fetal stage, it is characterized by the activation of a regenerative process which results in the complete restoration of the native structural and functional tendon properties occurring without any deposition of intermediate scar/fibrous tissue (16, 17).

Based on the fragmentary information collected to date, fetal and adult tendon differences in healing properties might depend on a complex interaction between the mechanisms involving both local and systemic conditions. Local conditions that may be responsible for the progressive reduction of tendon healing involve changes in the precursor cell niche, the activities of cell-intrinsic pathways (16, 18–20), and the reduction of paracrine response to tissue injury (21–23). Furthermore, different levels of key growth factors and cytokines have been identified from early gestation to the post-natal period, which could explain the age-related scarless reparative phenotype (22).

Overall, these findings may explain the increased cellular migration, tenocyte synthetic activity (24–30), and cell-to-cell communication observed in fetal tissues (25–35), implying that the greater plasticity of the fetal tendon is required to express a high healing performance (12), even though the conclusive mechanisms remain unknown.

As a result, understanding the mechanisms involved in early successful tenogenesis and the related key molecular pathways is critical to innovate tendon diagnosis and therapy based on solid biological foundations.

Because of these needs, it is relevant to stress that animals play a triple role in each field of tendon biomedicine: patiently awaiting clinical solutions, predictive models which collect experimentally the tissue stage-specific biological information, and, finally, the translational role supporting indirectly medical clinic advances.

Several animal models, in particular, are recognized to have a clinical translational value in addition to allowing researchers to investigate regenerative mechanisms at an early stage of life when biomechanical and physiological tendon properties are still preserved. Considering fragmentary clinical data in adulthood, these models must be exploited to obtain information related to factors, molecules, and signaling pathways with key roles in driving the proper regeneration of tendon tissue. Nevertheless, these molecules may potentially offer clinical therapeutic solutions to enhance tissue regeneration avoiding unsuccessful adaptive reparative processes.

Based on these premises, the present research has been designed to map the genetic factors underlying tendon development during embryo/fetal and pre-puberal life stages identified to date to generate either literature or enriched-derived data collections. This systemic molecular survey was the premise to decipher tendon biology by exploiting computational biology to generate species-specific networks.

The Tendon NETworks offered a data-driven computational framework based on molecules (upstream, transcription factors, and downstream levels) and a stage-stratified (fetal vs. prepubertal) model useful to identify the main players of tenogenesis. In addition, taking advantage of computational network enrichment, it was possible to decode signaling cascades and physiological paths driving toward an efficient and complete tenogenesis in early-stage organisms.

Overall, this study highlights the value of mapping complex and partially known biological processes using comparative systems biology. At the same time, the obtained results confirm the role of systems biology in making more comprehensive the evidence collected to date and, what is more important, in proposing new nodes and pathways to promote biology advances potentially impacting clinics.

## 2. Materials and methods

### 2.1. Data collection retrieval and organization

The bibliographic data collection was carried out following the Preferred Reporting Items for Systematic Review and Meta-analysis (PRISMA) Statement 2020 Checklist Guidelines.^1^ Scientific literature published in the past 30 years in the peer-reviewed international index Advanced Search of Web of Science [v.5.35] “Core collection” archive^2^ was considered.

A combination of the following keywords was adopted: “tendon,” “biology,” “tenogenesis,” “fetal,” “fetus,” “develop,” “mechanism,” and “pathway.” The words “injury,” “disease,” “rupture,” “inflammation,” and “tendinopathies” were excluded from the research. “TS” was used as a Field tag, “AND,” “OR,” and “NOT” were used as Boolean operators, and * as wild cards.

In total, 2,332 publications were retrieved including original articles and reviews, and duplicates were removed. Filtering criteria adopted included the following: (I) selection of original articles written in English; (II) original papers concerning the early stage of tendon development during embryo–fetal and pre-puberal life were exclusively considered; (III) original articles regarding tenogenic differentiation of stem cells and/or related to regeneration or pathology were discarded; and (IV) reviews were not included in the data collection; however, their content was examined to better support and discuss the acquired data. Finally, 37 publications of original scientific manuscripts describing molecular interactions including direct (physical) and indirect (functional) association met the inclusion criteria (Supplementary File 1) and those constituted the final data collection considered to build the species-specific tenogenesis networks (TendonNETs). This methodological procedure was successfully applied and previously validated by our group (36, 37). In detail, molecular interactions including physical and functional associations have been depicted as follows in the data collection. Each interaction has an input molecule that begins the signal and a target molecule that receives the signal. The signal could be mediated by physical interaction allowing the deposition of post-translation protein modifications regulating protein activity and/or genome accessibility. Moreover, the signal could be mediated by the functional association in the case of studies that use correlative data supporting upregulation and/or downregulation of the activity of the target molecule dependent on the presence and/or absence of the input molecule. The type of signal transmitted has been referred into the data collection sheet as a link (Supplementary File 2). Microsoft Excel (file .xls /Version 16.71/23031200) was used for data collection.

### 2.2. Tenogenesis network creation, visualization, and analysis

**Figure 1:**
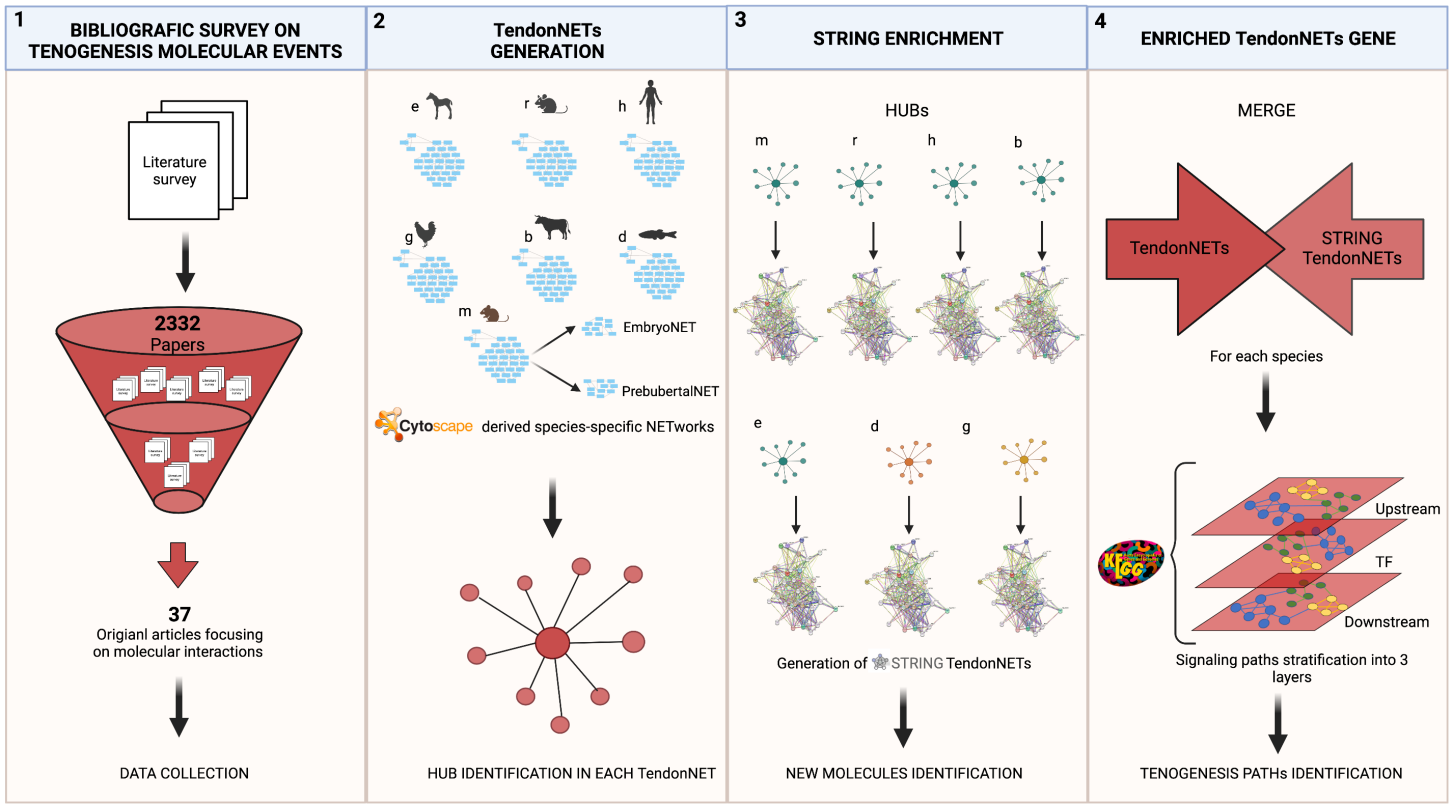
The methodological flow of the study. The study recognizes a stepwise approach focused on four main tasks: (1) The bibliographic survey performed by accessing Web of Science collection related to tenogenesis molecular events aimed to the selection of pertaining publications and data collection; (2) TendonNET generation was based on literature evidence and aimed to build up species-specific interaction networks supporting tenogenesis and to identify hubs (hyperlinked nodes) of each species-specific network. Species-specific networks and hub identification were obtained by using the Cytoscape tool. Only data collected from the Mus musculus (m) were also processed by distinguishing networks of interactions occurring during the embryo and prepubertal developmental phase; (3) the hubs of each species-specific TendonNETs were used as input for the generation of networks of new interactions of the tenogenesis (STRING TendonNETs) by using the STRING tool; (4) finally, combining interactions collected by literature evidence (TendonNETs) with potential new interaction (STRING TendonNET), species-specific enriched networks (Enriched TendonNETs) were generated. The organization of signaling events was then investigated by stratifying signaling paths supporting tenogenesis into three interconnected layers, such as upstream, processing TF (Transcription factors), and downstream. Specific abbreviations were used for each model organism (otherwise referred to as species). Mus musculus (m), Rattus norvegicus (r), Homo sapiens (h), Equus caballus (e), Bos taurus (b), Gallus (g), and Danio rerio (d). Created with BioRender.com.

Data collection referred to each model organism was used as input to build a species-specific tenogenesis network using the Cytoscape 3.9.1 software.^3^ Seven networks were obtained. Each species-specific network was then analyzed with the dedicated plug-in Network Analyzer of Cytoscape by computing the following topological parameters: number of nodes, number of edges, the average number of neighbors, network diameter, network radius, characteristic path length, clustering coefficient, network density, and connected components. Additional analyses applied to networks and nodes have been listed in the following sub-sections. Microsoft Excel (file .xls /Version 16.71/23031200) was used for data visualization and/or analysis. Detailed methodological procedures supporting the present study are reported in Figure 1.

#### 2.2.1. Identification of hyperconnected (hub) nodes

The hubs, defined as hyperconnected nodes, were identified as nodes with a degree of at least one standard deviation above the network mean as previously described (37–39).

#### 2.2.2. Node degree

Each network node was ranked based on the node degree value. Node degree is defined as the number of connections that the node has to other nodes in the network (7).

#### 2.2.3. MCL and local network clustering

The Markov Cluster Algorithm (MCL), based on the simulation of a stochastic flow in graphs, was computed to define clusters of highly related nodes in each analyzed network flow. Clustering data were obtained by setting 3 as the inflation parameter. A local network clustering algorithm was applied to each MCL-derived cluster to measure control over information flows between just the immediate neighbors of the cluster. MCL and local network clustering algorithms were supplied by the Search Tool for the Retrieval of Interacting Genes/Proteins (STRING) platform (STRING).^4^

#### 2.2.4. STRING enrichment analysis

A Search Tool for the Retrieval of Interacting Genes/Proteins (STRING, see text footnote 4) (40) was used to enrich the data collection by including known and predicted protein interactions. They could be either direct (physical) or indirect (functional) associations and were derived from different sources: genomic context, high-throughput experiments, conserved coexpression, and previous knowledge. A new network was obtained by adopting a medium confidence score (0.400). For the enrichment procedures, the false discovery rate (FDR) value was set to be <0.05, and three cycles of enrichment were performed.

#### 2.2.5. KEGG analysis

Pathways characterizing the tenogenesis network were retrieved from the Kyoto Encyclopedia of Genes and Genomes (KEGG). KEGG is a database resource for understanding high-level functions and utilities of the biological system, and from molecular-level information, especially large-scale molecular datasets generated by genome sequencing and other high-throughput experimental technologies.^5^

#### 2.2.6. Venn diagram

A Venn diagram tool^6^ was used to visually represent the differences and similarities among species-specific tenogenesis networks.

## 3. Results

### 3.1. A multi-organism data collection strategy to depict the backbone of the tenogenesis signaling events

To compose the knowledge regarding tenogenic molecular events, evidence-based data including molecular interactions or signaling events (hereinafter referred to as interactions) were collected from the available literature (Supplementary File 1) to build up a data collection encompassing the interspecific tenogenesis pathways (Supplementary File 2).

**Figure 2:**
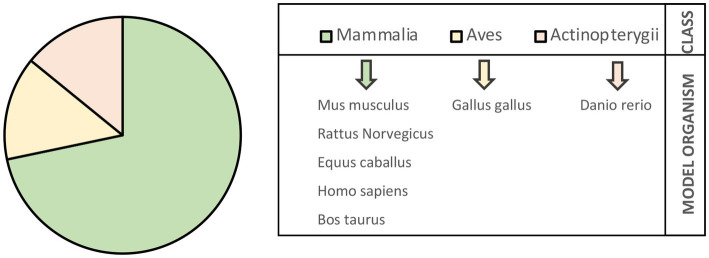
Model organisms identified by collecting literature data on signaling events in embryonic and prepubertal tenogenesis.

First, the interactions were collected according to the model organism and classified based on the developmental stage of occurrence (embryo–fetal and prepubertal stage). The molecular data were retrieved from seven model organisms, most of them belonging to the Mammalia class: Mus musculus (m), Rattus norvegicus (r), Homo sapiens (h), Equus caballus (e), and Bos taurus (b). Two additional models were represented belonging to the Aves and Actinopterygii classes, respectively: Gallus gallus (g) and Danio rerio (d) (Figure 2).

Among Mammalia class, half of the interactions belong to m (145/286; 51%), approximately 10% to r and e (28 and 28 out of 286, respectively), 3% to h (9/286), and a minority of them to b (2/286; 0.7%).

Interactions retrieved from g accounted the 25% (70 out of 286), whereas d contributed only 1.4% (4/286) (Table 1).

**Table 1:** Classification of interactions collected from literature and classified according to the organism model and the stage of development (embryo or prepubertal stage).

Class	Model organism (acronymus)	Interactions		
		Total edges	Developmental stage	
			Embryo	Prepubertal
Mammalia	Mus musculus (m)	145	71	74
	Rattus norvegicus (r)	28	0	28
	Homo sapiens (h)	9	9	0
	Equus caballus (e)	28	3	25
	Bos taurus (b)	2	0	2
Aves	Gallus gallus (g)	70	68	2
Actinopterygii	Danio rerio (d)	4	4	0
Total interaction numbers		**286**	**155**	**131**

The acronyms of each model organism were indicated as m for Mus musculus, r for Rattus norvegicus, h for Homo sapiens, e for Equus caballus, g for Gallus gallus, and d for Danio rerio.

The data collection was also designed to distinguish between embryonic (155 out of 286; 54%) and prepubertal (131/284; 46%) vital stages (Table 1).

**Figure 3:**
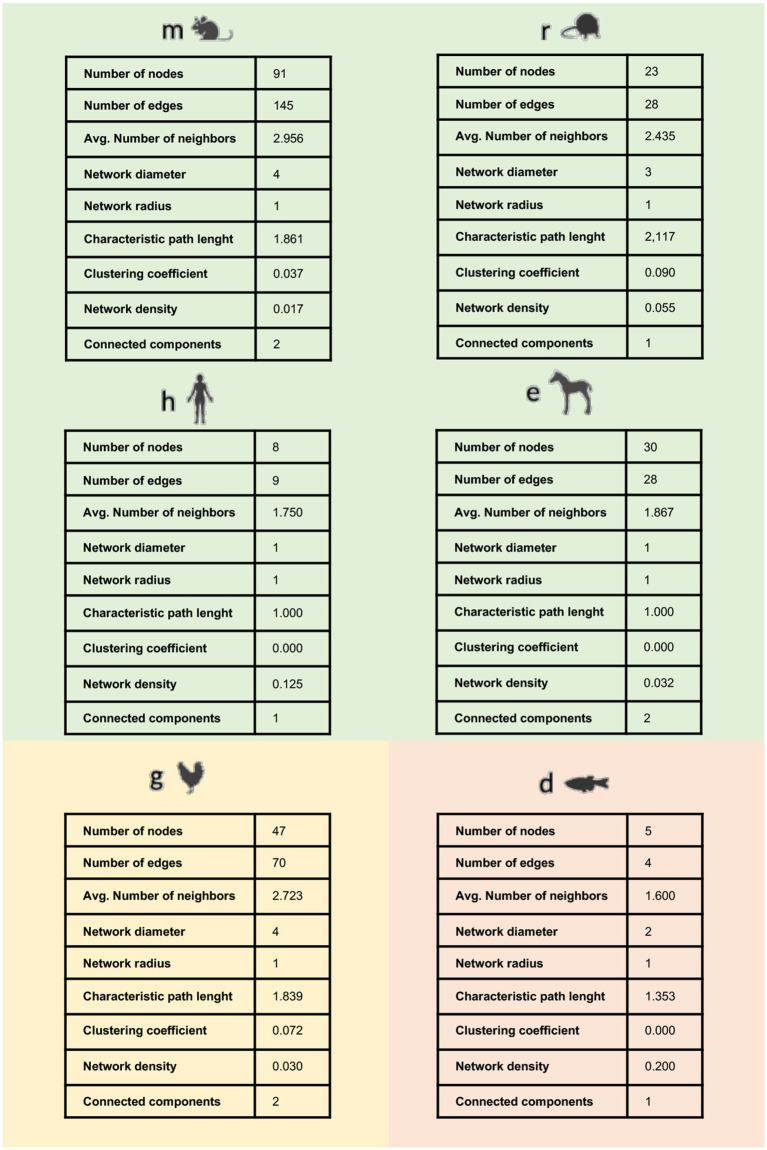
Main topological parameters of species-specific TendonNETs. Topological analysis was not accomplishable for the Bos taurus dataset based on the limited number of retrieved nodes.

Second, the set of interactions identified in each model was used as input to generate a species-specific system network (hereinafter collectively referred to as TendonNET identified through the species-specific acronymous), whose topological parameters were computed as reported in Figure 3 and Supplementary File 3. The TendonNETs independently of the species displayed a scale-free topology with a very low clustering coefficient value, according to the Barabási-Albert (BA) model.

Third, nodes composing each TendonNETs were assembled using the MCL-clustering algorithm^7^ to define the main paths of the molecular flow and to depict what is currently known about them in each model organism.

The MCL approach enabled us to classify 12 clusters in the mTendonNET, 3 of them in the gTendonNET and 2 in dTendonNET and hTendonNET, whereas both rTendonNET and eTendonNET recognized exclusively 1 cluster (Table 2). Then, paths characterizing each TendonNET cluster were identified by using the local algorithm supplied by the STRING platform (see text footnote 4) and selected by applying the False Discovery Rate (FDR) value of <0.05 (Table 2 and Supplementary File 4). Coherent tissue-specific paths were identified. In detail, m, h, and eTendonNETs shared target molecules of collagen formation paths. Consistently, tendon-related paths were highly conserved (m, r, h, and d), as well as TGFB signaling (m, g, and r) and extracellular matrix (ECM) organization (m and gTendonNETs, respectively). Of note, neuronal-related pathways were also widespread: NGF appears among the GFs in mice path while an entire neuronal path (neuronal-related process: microglia cell activation) has been recognized in e. Probably, because of the early-stage development of the selection of molecules, skeletal muscle-related paths were also identified such as bone formation and skeletal muscle fiber development in r and d, respectively. Growth and development paths involved common molecules (in d, r, and m) such as insulin, vascular, and hedgehog.

**Table 2:** Tenogenesis network flow definition by MCL-clustering algorithm.

Tenogenesis networks	MCL cluster numbers	Main MCL recognized paths
mTendonNET	12	Collagen fibril and keratan sulfate biosynthesis and degradation ECM organization Tendon formation Growth factor (NGF, TGFB, IGF, VEGF, and Hedgehog) signaling
rTendonNET	1	Tendon formation Bone formation TGFB signaling
hTendonNET	2	Tendon formation Collagen formation
eTendonNET	1	Collagen formation Neuronal-related process (microglia cell activation)
bTendonNET	No significant enrichment detected	
gTendonNET	3	TGFB and BMP signaling ECM organization
dTendonNET	2	Tendon formation Skeletal muscle fiber development

The number of clusters retrieved in each TendonNET was classified for their main paths. Clustering data were not generated for the b TendonNET for the limited number of interactions. FDR was set at <0.05 value. For detailed information, see Supplementary File 4.

**Figure 4:**
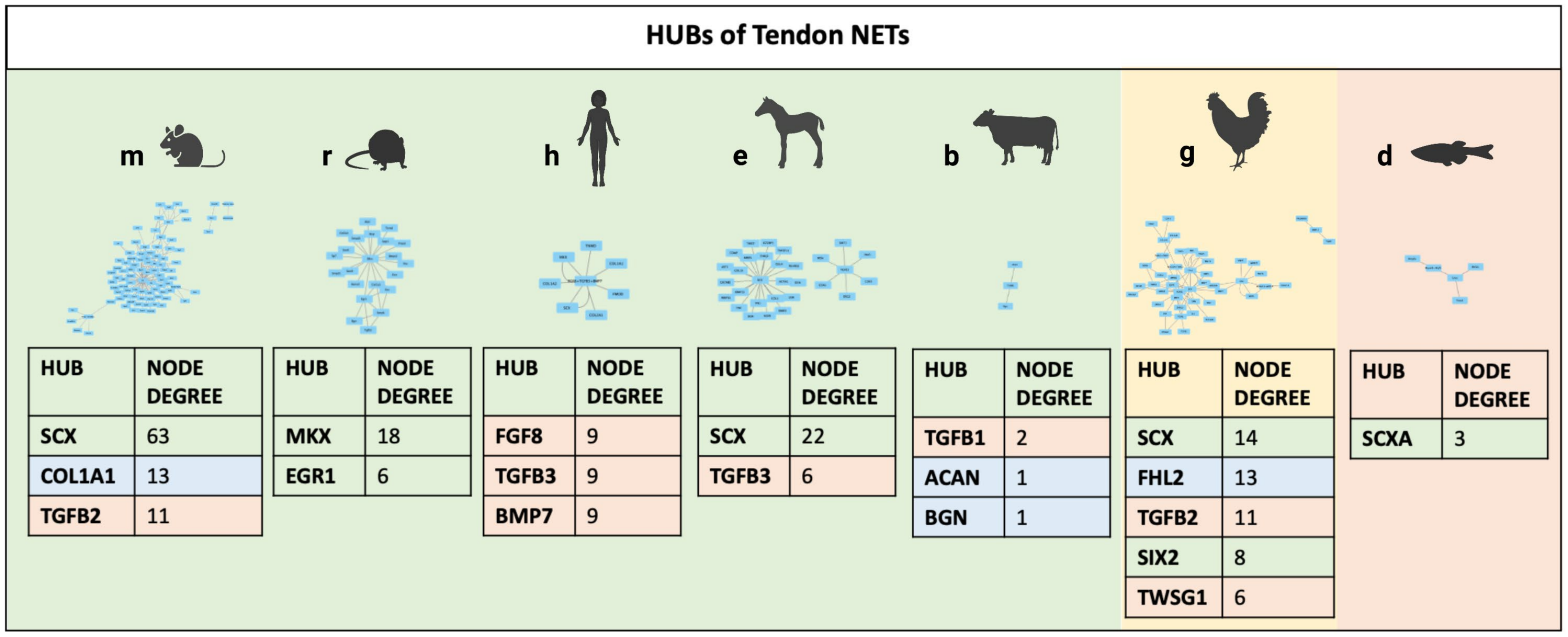
Hubs of TendonNETs. Hubs were identified and ranked based on the hub node degree value. The different colors of the hub molecules allow to identify the signaling layer of pertinence. In orange were reported nodes of the upstream layer; in green nodes of the TF layer, and in light blue nodes of the downstream layer.

Finally, to identify and prioritize the influential molecules of each TendonNET, hyperconnected nodes, defined as hubs, were identified, and ranked based on their node degree value (Figure 4). On the contrary, the bottleneck was not accomplished because of the small number of interactions in each TendonNET.

Among the 14 hubs identified, upstream molecules belonging to the TGFB family and key TF (TF), such as SCX, MKX, FHL2, and SIX 2, have been recognized. Of note, SCX and members of the TGFB superfamily (TGFB1, TGFB2, TGFB3, and TWSG1) appeared to be highly conserved since they are shared in four different model organisms (Figure 4).

### 3.2. Tenogenesis signaling identified by TendonNETs is strictly developmental stage-dependent

To provide timing to the identified molecular interactions of mTendonNET, the nodes were further classified on the bases of the developmental stage to build two stage-specific NETs: embryo TendonNET (including interactions of both embryo and fetal stages) and prepubertal TendonNET. This analysis was carried out exclusively for the mouse model since it only offered enough interactions in both vital stages.

**Figure 5:**
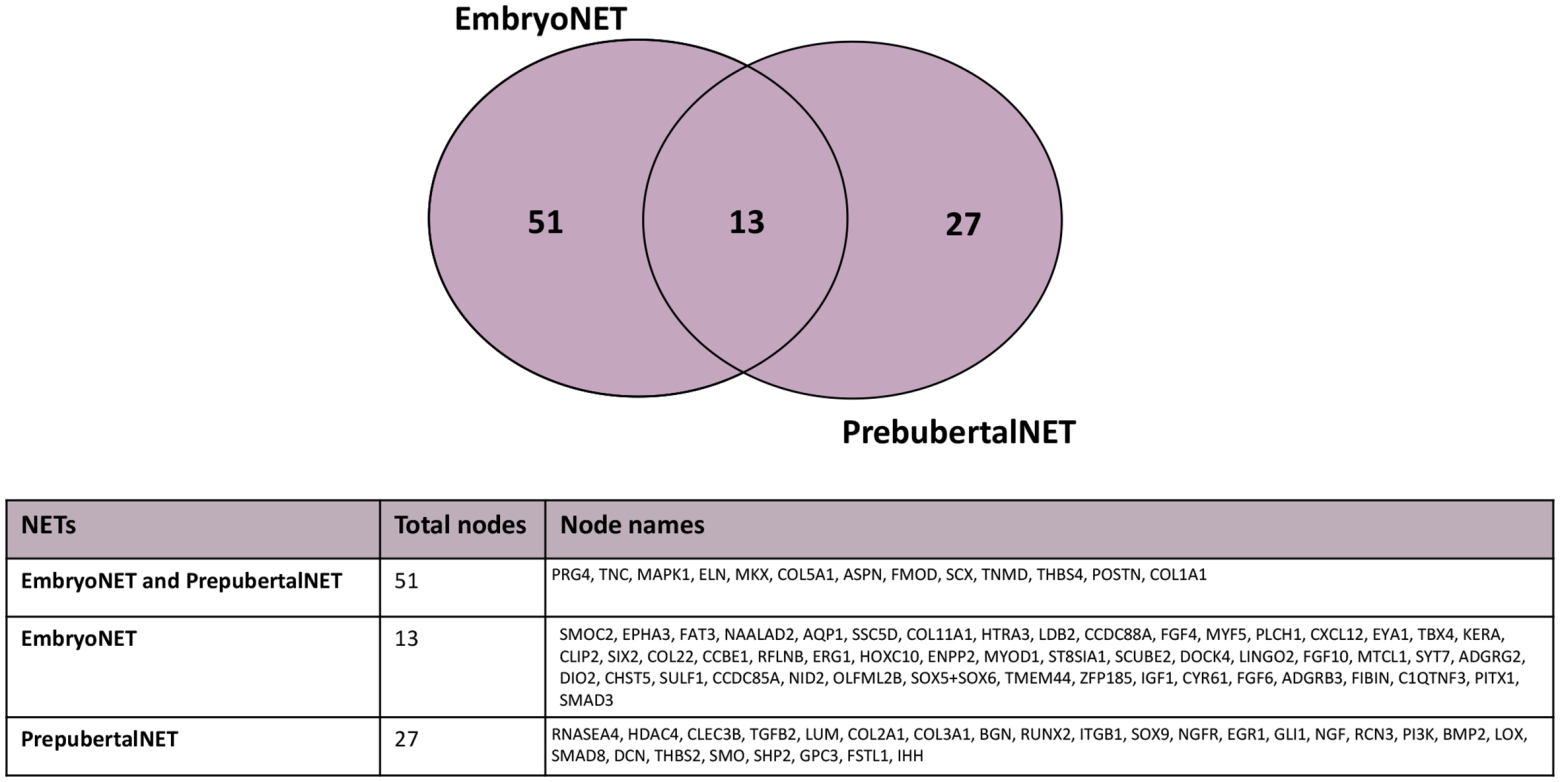
Venn diagram representing the shared nodes of the embryo TendonNET and the prepubertal TendonNET of m model organism. The classification of nodes in the embryo or prepubertal category was defined during the data collection (Supplementary File 2).

A low overlapping degree (14%; 13 nodes) was found among the nodes composing embryo TendonNET (64 molecules) and prepubertal TendonNET (40 molecules) (Figure 5).

**Figure 6:**
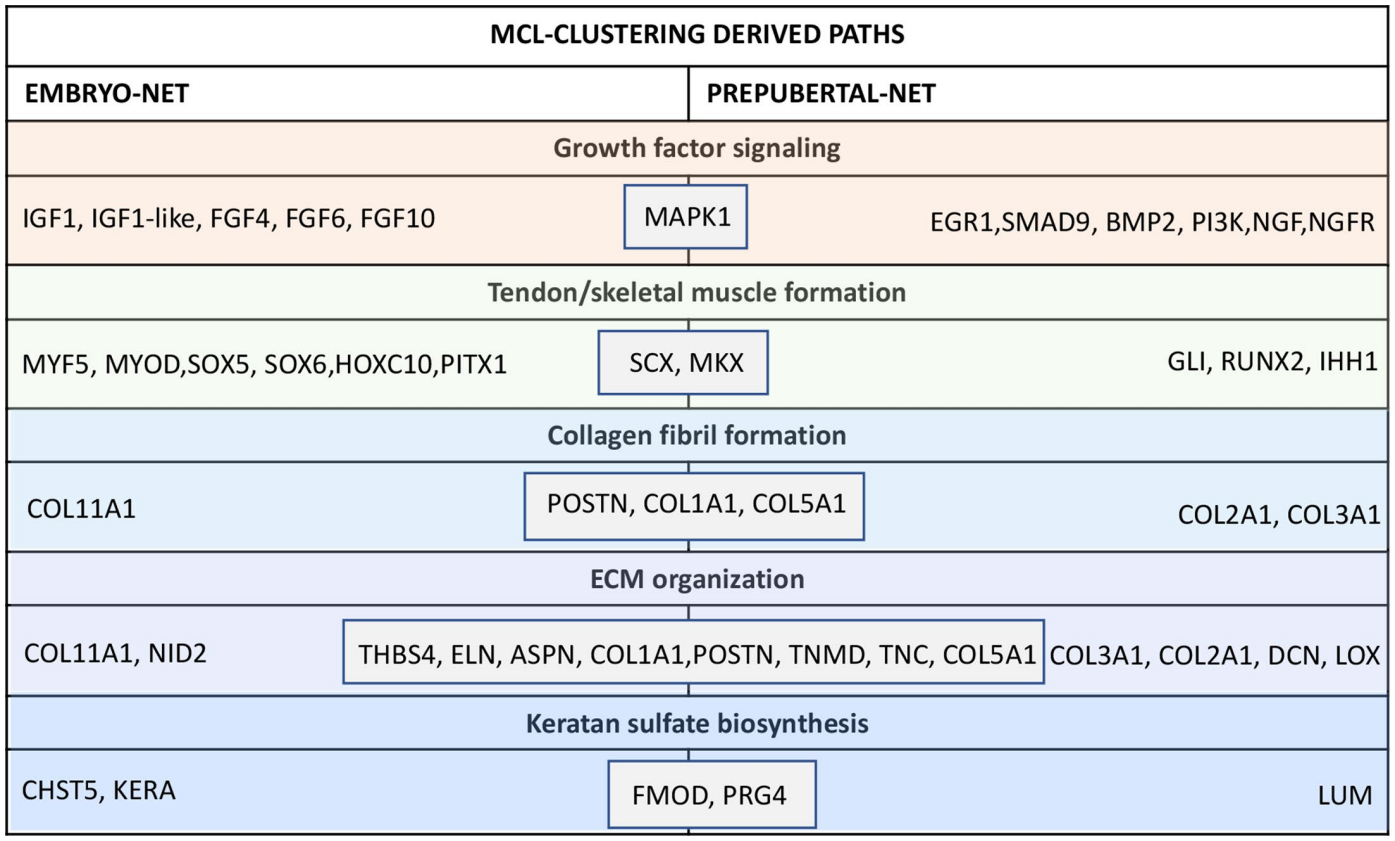
Nodes clustering of the mouse embryo and prepubertal TendonNETs. The table summarizes the results of clustering classified for MCL-recognized paths by indicating within the gray boxes the 13 shared nodes. In orange were reported nodes of the upstream layer, in green nodes of the TF layer, and in light blue nodes of the downstream layer.

Interestingly, their MCL-clustering algorithm showed that these 13 nodes belong to common paths in both embryo or prepubertal TendonNETs (Collagen fibril formation, ECM organization and Keratan sulfate biosynthesis, growth factor signaling, and tendon skeletal muscle formation) even if they take part in different interactions that appeared to be strictly vital stage-dependent (Figure 6 and Supplementary File 5).

More in detail, the collagen fibril formation path recognized POSTN, COL1A1, and COL5A1 as shared molecules which clustered with COL11A1 in the embryoTendonNET or with COL3A1 and COL2A1 in the prepubertal TendonNET. Similarly, the ECM organization path shared THBS4, ELN, ASPN, COL1A1, POSTN, TNMD, TNC, and COL5A1 clustering with COL11A1 and NID2 in the embryo TendonNET or with COL3A1, COL2A1, DCN, and LOX in the prepubertal TendonNET.

Furthermore, FMOD and PRG4 were shared nodes of the Keratan sulfate biosynthesis and clustered with CHST5 and KERA in the embryo TendonNET and with LUM in the prepubertal TendonNET.

Tendon/skeletal muscle formation path shared the transcription factors (TF) SCX and MKX clustering with MYF5, MYOD, SOX5, SOX6, HOXC10, and PITX1 in the embryo TendonNET and with GLI1, RUNX2, and IHH1 in the prepubertal TendonNET.

Concerning the growth factor signaling path, MAPK1 was identified as a shared node clustering with IGF1, FGF4, FGF6, and FGF10 in the embryo TendonNET and with EGR1, SMAD8, BMP2, IGF downstream molecules PI3K, and nerve growth factor signaling molecules NGF and NGFR in the prepubertal TendonNET.

Furthermore, the topological properties of the embryo and prepubertal TendonNETs were computed allowing to define the relative hubs, which also showed a limited level of overlapping. More in detail, the tendon-specific transcription factor SCX was the unique hub of the embryo TendonNET that resulted even to be a shared node. A higher number of hubs was identified in the prepubertal TendonNET. They recognized upstream molecules (TGFB1 and NGF), TF (SCX), and downstream nodes (COL1A1, BGN, signaling pathway-related molecules SHP2, and hedgehog SMO: Table 3 and Supplementary File 6). Overall, the different interconnections depicted by the two NETs’ topological parameters reflect the diverse mechanisms underlying tissue organization during the embryo and early post-natal stages.

**Table 3:** Hub molecule identification and ranking in embryo TendonNET and prepubertal TendonNET.

HUB				Embryo TendonNET	PrebubertalNET	Hub	Node degree	Hub	Node degree
SCX	55	SCX	12
		TGFB2	11
		SMO	10
		SHP2	10
		COL1A1	9
		BGN	7
		NGF	7

The ranking was based on the value of the hub node degree.

### 3.3. Knowledge-based network enrichment identifies new molecular players of the tenogenesis process working in a multi-layered signaling system

To overcome the main limitation due to the small number of evidence-based data concerning the molecular interactions in tenogenesis, the hubs identified in species-specific TendonNETs were used as input for seeking new functional protein associations by using the STRING tool.

Based on the limited number of interactions collected for the b, all nodes were used as input for further analyses.

Functional enrichment allowed the generation of string-derived species-specific networks (hereinafter referred to as StringNETs) bearing new nodes predicted to be associated with signaling pathways supporting tenogenesis (Supplementary File 7).

**Figure 7:**
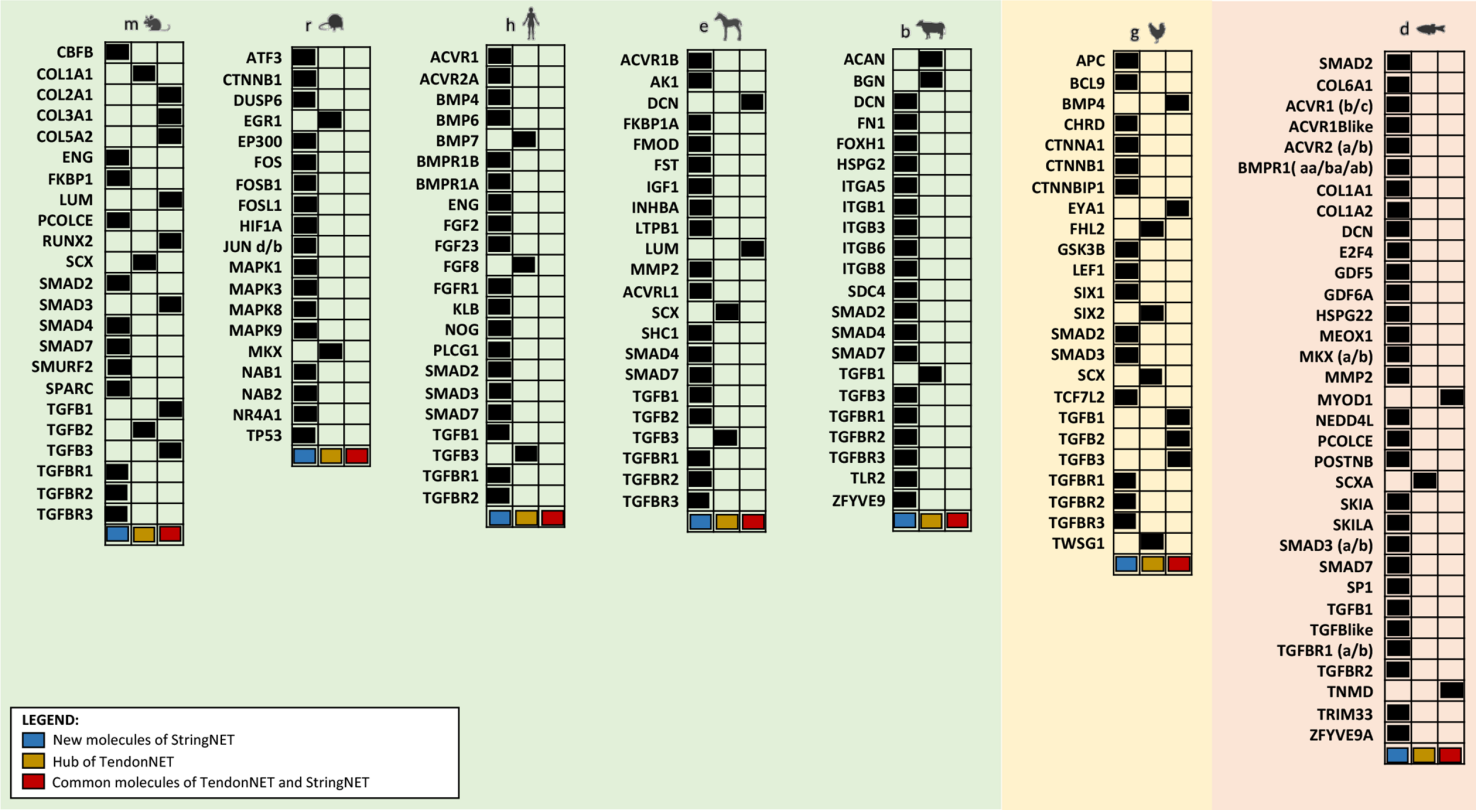
Overview of nodes composing StringNETs. StringNETs were obtained using the STRING tool (https://string-db.org/) with the hubs of the TendonNETs as input except for b where the few nodes identified in TendonNET were used. The classification presented in the figure was aimed to distinguish inside each model organism the hub molecules of TendonNETs (in yellow), the StringNETs enriched nodes (in light blue), and the common nodes between TendonNETs and StringNETs (in red).

Specifically, 77 out of 99 nodes characterizing StringNETs were new (Figure 7).

First, all nodes composing the StringNETs were stratified into three main signaling layers:

- Upstream molecules enclosing growth factors and their related intracellular transduction factors. In total, there were 52 molecules, of which 38 were newly identified by STRING (73%).
- TF-bearing skeletal muscle and neuronal-related TF, homeobox genes, oncogenes, and chromatin remodelers. In total, there were 23, of which 15 were newly defined by STRING (65%).
- Downstream effectors include 24 molecules involved in the ECM organization, neuronal cytoskeleton factors, and cell adhesion and binding activities. In total, there were 24, of which 15 were identified as new by STRING (62%).

**Figure 8:**
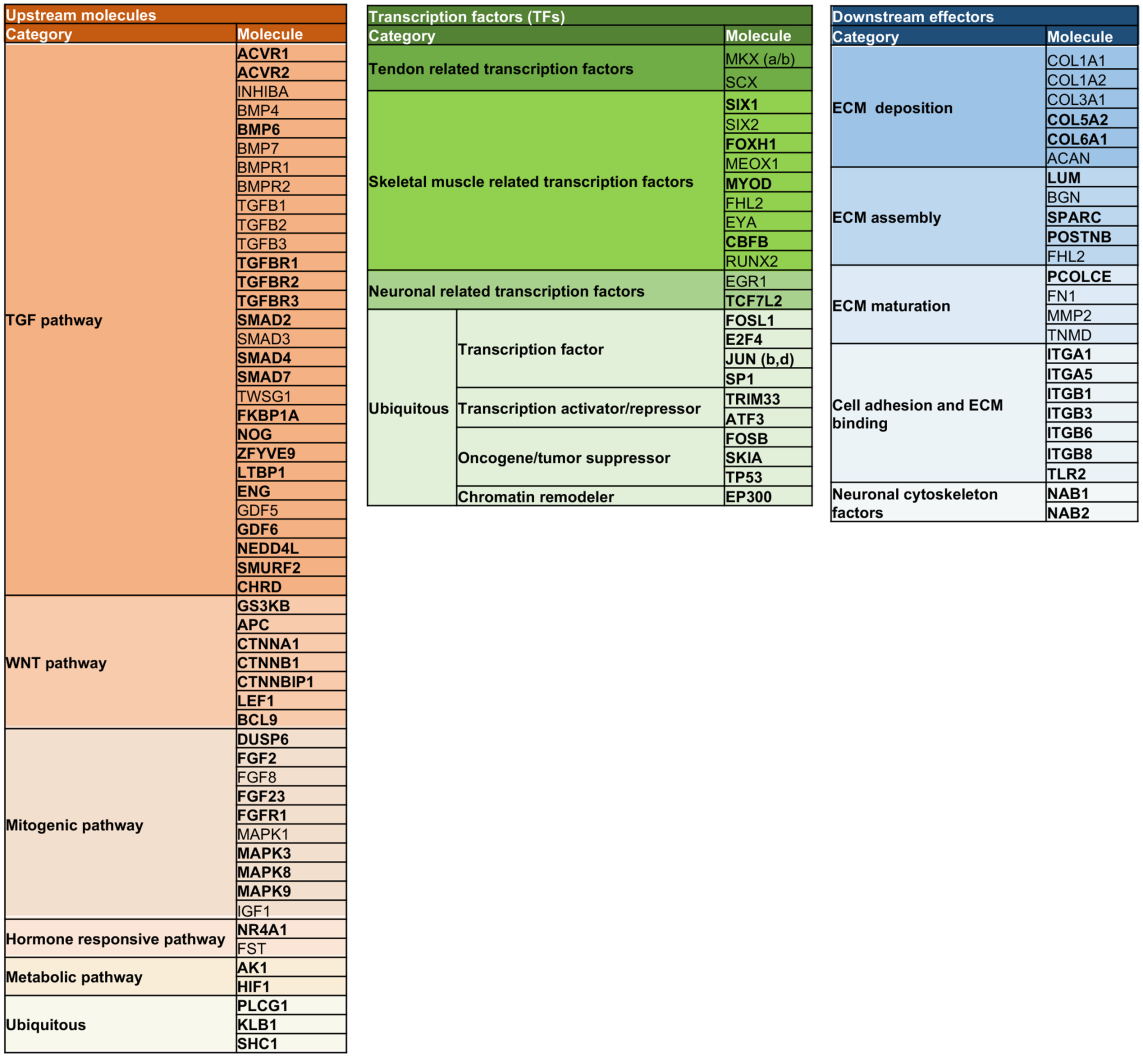
Multi-layered organization of the overall molecules composing the TendonNETs and the StringNETs. New molecules identified by STRING have been reported in bold. The different colors of the nodes allow to identify the signaling layer of pertinence. In orange were reported nodes of the upstream layer; in green nodes of the TF layer, and in light blue nodes of the downstream layer.

The stratification analysis allowed to depict a model composed of molecules working at different layers of the signaling events primed by extra cell stimuli and conveyed by cytoplasmic signaling molecules toward the activation of tendon/skeletal muscle-critical TF, which are responsible for priming tendon/skeletal muscle development and organization (Figure 8).

More in detail, the stratification enabled us to depict connections among the three identified layers of the tenogenic signaling as follows.

The signal starts with molecules characterizing the upstream layer that included growth factors and intracellular components belonging to the TGF (ACRVRs, TGFBs, TGFBRs, BMPs, SMADs, TWSG1, GDFs, and LTBP1), WNT (CTNNA, CTNNBs, LEF, APC, and GSK3B), mitogenic (MAPKs, DUSP6, FGFs, and IGFs), hormone-responsive (NR4A1, FST, and BCL9), and metabolic (HIF1 and AK1) pathways. Signaling converges on the intracellular transcriptional machinery recognizing tendon (SCX, MKXs, EYA, and SIXs), muscle skeleton (FOXH1, MEOX, MYOD, CFBF, and RUNX2), and neuronal (EGR1 and TCF7L2) TF. The transcriptional program is addressed to modulate the expression of the extracellular matrix (ECM) assembly (LUM, BGN, SPARC, POSTNB, and FHL2), maturation (PCOLCE, FN1, MMP2, and TNMD), and adhesion (ITGAs, ITGBs, and TLR2), as well as molecules constituting the essential structural blocks of the ECM (COL1, COL3, COL5, COL6, and ACAN) could be out lighted as the functional outcomes of the signaling cascade.

### 3.4. *Enriched* TendonNETs predict intriguing new hubs of tenogenesis signaling

To unveil new potential branches of the tenogenesis signaling, all the nodes identified in both TendonNET and StringNET data collections were merged using Cytoscape to generate species-specific Enriched TendonNETs (Supplementary File 8). Kyoto Encyclopedia of Genes and Genomes (KEGG) mapping tool was then applied, respectively, to the nodes of TendonNETs and Enriched TendonNETs (Supplementary Files 9, 10).

KEGG paths were grouped into macro-categories according to the BRITE hierarchy criteria^8^ (see, respectively, Supplementary Files 9, 10) and those related to the physiology of tendon annotated as shown in Table 4.

**Table 4:** KEGG paths grouped into BRITE macro-categories related to TendonNETs and *Enriched* TendonNETs.

NETs	TendonNETs		EnrichedNETs	
	Physiological and diseaseKEGG paths number	Macro-categories of physiological KEGG paths	Physiological and disease KEGG paths number	Macro-categories of physiological KEGG paths
m	46	Growth, differentiation, and survival (12) Morphogenesis and cell motility (5) Nervous system (2) Endocrine system (2)	56	Growth, differentiation, and survival (15) Morphogenesis and cell motility (6) Nervous system (2) Endocrine system (2)
r	10	Growth, differentiation, and survival (5) Morphogenesis and cell motility (2)	126	Growth, differentiation, and survival (29) Morphogenesis and cell motility (9) Nervous system (10) Endocrine system (12)
h	7	Growth, differentiation, and survival (2) Morphogenesis and cell motility (2)	38	Growth, differentiation, and survival (13) Morphogenesis and cell motility (4) Nervous system (1) Endocrine system (1)
e	11	Growth, differentiation, and survival (1) Morphogenesis and cell motility (2) Endocrine System (1)	36	Growth, differentiation, and survival (8) Morphogenesis and cell motility (2) Endocrine system (1)
b	–	n.d.	49	Growth, differentiation, and survival (13) Morphogenesis and cell motility (6) Endocrine system (1)
g	5	Growth, differentiation, and survival (3) Morphogenesis and cell motility (1) Endocrine System (1)	10	Growth, differentiation, and survival (4) Morphogenesis and cell motility (5) Endocrine system (1)
d	–	n.d.	10	Growth, differentiation, and survival (7) Morphogenesis and cell motility (3)
Total unique paths count*	50	23	142	62

*The number of total paths retrieved was counted excluding duplicate paths in the different NETs. See Supplementary File 11, columns “Total unique physiological path count” and “Total unique path count” for further details.

Based on the collected data, KEGG applied to TendonNETs allowed us to draw the backbone of the signaling supporting tenogenesis. KEGG analysis of the Enriched TendonNETs allowed to enrich the network map defining new paths and/or network links. This was clear by looking at the increased number of paths recognized in each macro-categories for each NET. Also, the total physiological number of paths retrieved, respectively, in TendonNETs and Enriched TendonNETs reflected this aspect. Indeed, physiological paths recognized in TendonNETs (23) are triplicated when compared with paths of the Enriched TendonNETs (62) (Supplementary File 11). Also, the analysis unveiled biological coherent paths shared among different model organisms, specifically identifying in three out of seven species, upon enrichment, paths belonging to BRITE macro-categories such as (1) growth, differentiation, and survival, (2) morphogenesis and cell motility, (3) nervous system, (4) and endocrine system. All paths are recognized by the literature-based data of Mus musculus, the best-characterized model organism. Importantly, enrichment allowed a consistent representation of macro-categories that were under-represented in TendonNETs, including endocrine (retrieved in six species) and nervous (retrieved in three species) system-related paths.

**Figure 9:**
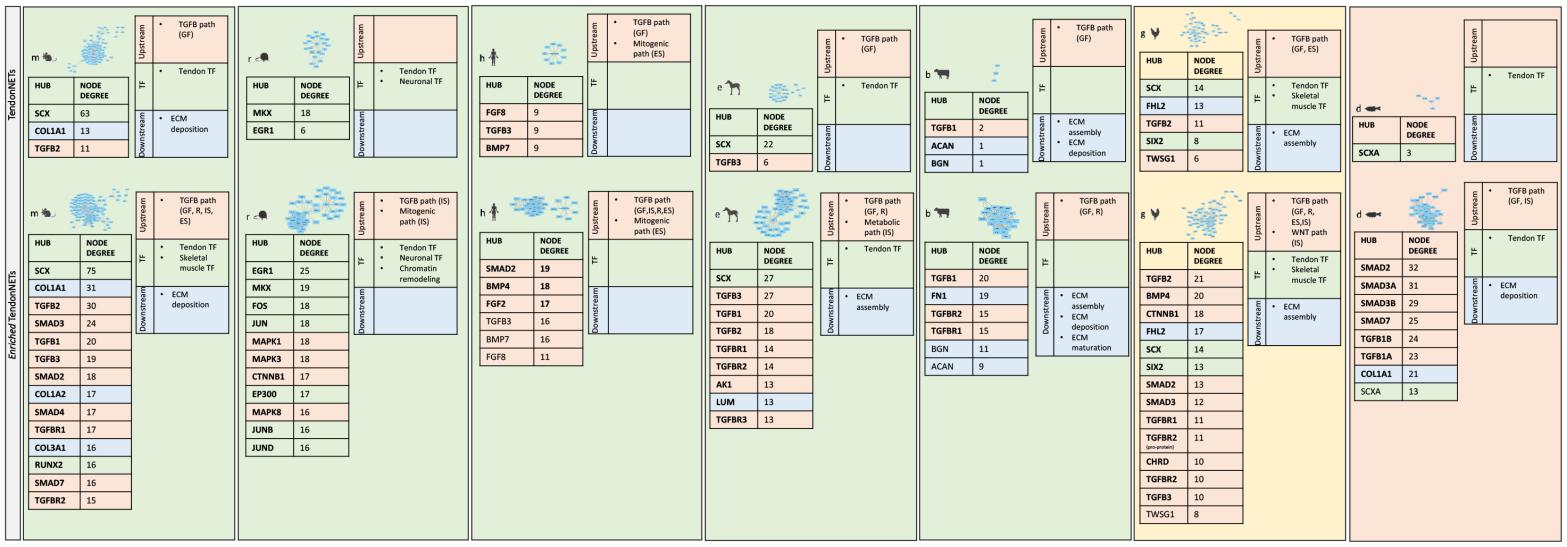
Hub identification and ranking in each *Enriched* TendonNET. The ranking was based on the value of the hub node degree. No bold font indicates molecules with a hub role in the TendonNETs (detailed in Figure 4). The different colors of the hub molecules allow us to identify the signaling layer of pertinence. In orange were reported nodes of the upstream layer, in green nodes of the TF layer, and in light blue nodes of the downstream layer. ES, extracellular signaling; IS, intracellular signaling; R, receptor; GF, growth factor.

Furthermore, topological analyses of the Enriched TendonNETs were performed to rank the hubs (Figure 9 and Supplementary File 12).

Interestingly, the topological analysis of the Enriched TendonNETs allowed us to better articulate the flow of information in tenogenesis by consistently increasing the number of hubs. In total, 25 new hubs were identified in the Enriched TendonNETs, which combine with the 14 outlined in the TendonNETs. Importantly, as highlighted in Figure 9, hubs of the Enriched TendonNETs nourished each layer of the tenogenic signaling with new key upstream, transcription, and downstream molecules and improved layer interconnections.

Moreover, the hubs retrieved into the upstream and TF layers of the tenogenic signaling of the different organisms confirmed a high degree of conservation (Table 5).

**Table 5:** Representation of the hubs of the *Enriched* TendonNETs organized into the multi-layered tenogenic signaling and classified for organism model.

Degree of inter specie-specific conservation of hubs of the *Enriched* TendonNETs classified into the three multi layered tenogenic signaling		
PATHs	HUBs	*Organism models*
Upstream molecules		
TGF path	TGFB1	m, e, b, d
	TGFB2	m, e, g
	TGFB3	m, h, e, g
	**SMAD2**	m, h, g, d
	**SMAD3**	m, h, g, d
	**SMAD4**	m
	**SMAD7**	m, d
	**TGFBR1**	m, e, b, g
	**TGFBR2**	m, e, b, g
	**TGFBR3**	e
	**BMP4**	h, g
	BMP7	h
	**CHRD**	g
	TWSG1	g
WNT path	**CTNNB1**	r, g
Mitogenic path	**MAPK1**	r
	**MAPK3**	r
	**MAPK8**	r
	**FGF2**	h
	FGF8	h
Metabolic path	**AK1**	e
TF		
Tendon/skeletal muscle TFs	SCX	m, e, g
	**RUNX2**	m
	MKX	r
	SIX2	g
Neuronal related TFs	EGR1	r
Ubiquitous	**JUN (b/d)**	r
	**FOS**	r
	**EP300**	r
Downstream effectors		
ECM maturation	**FN1**	b
ECM deposition	**COL1A1**	m, d
	COL1A2	m
	**COL3A1**	m
	ACAN	b
ECM assembly	BGN	b
	FHL2	g
	**LUM**	e

Data reported in this table derived from the application of the Venn diagram and stratification analyses to the hubs of the *Enriched* TendonNETs. The 24 new hubs derived after STRING enrichment are reported in bold. The different colors of the hub molecules allow to identify the signaling layer of pertinence. In orange were reported nodes of the upstream layer, in green nodes of the TF layer, and in light blue nodes of the downstream layer.

**Figure 10:**
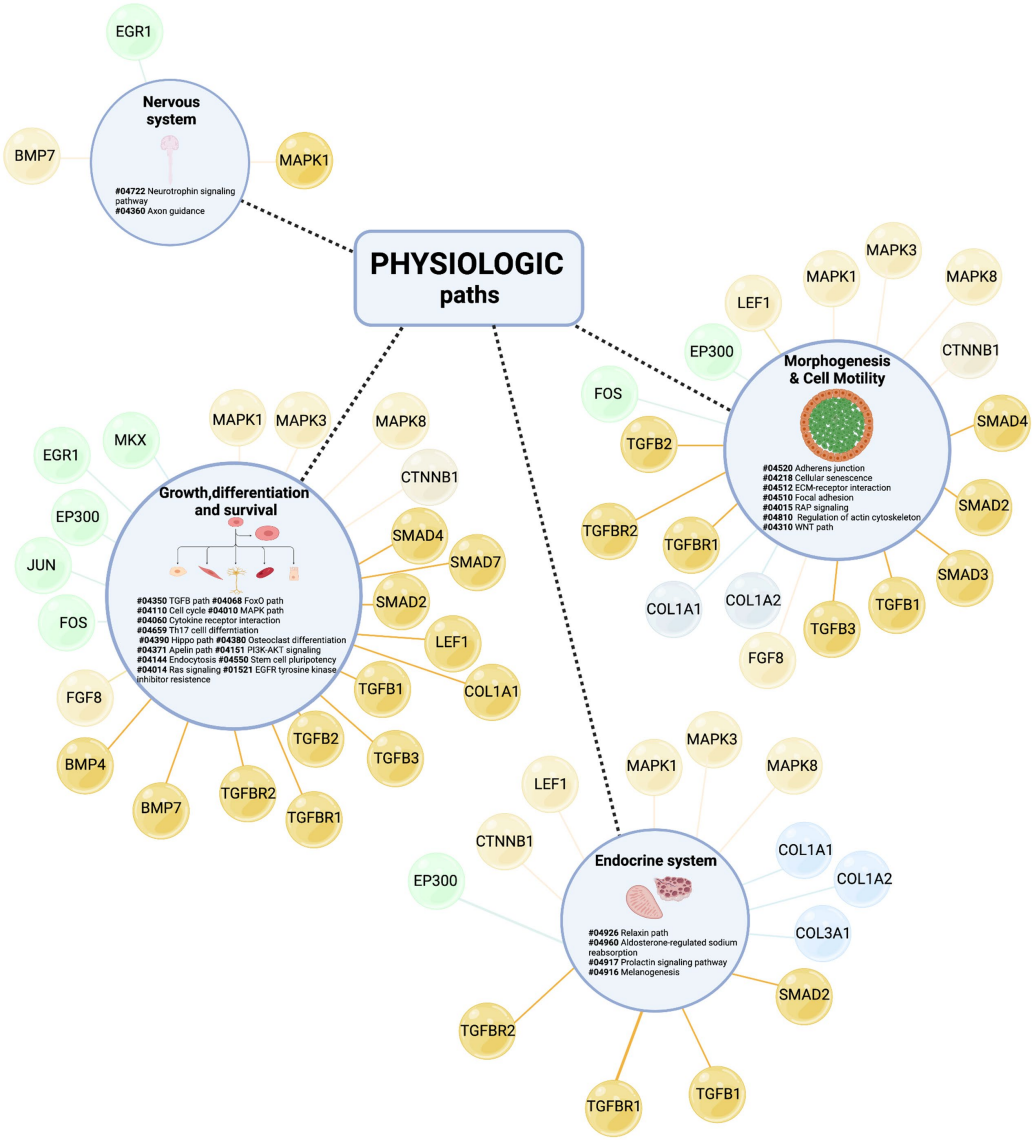
Identification of new paths sustaining tenogenesis signaling. Physiological-related BRITE macro-categories include (1) growth, differentiation, and survival, (2) morphogenesis and cell motility, (3) endocrine system, and (4) nervous system. For each macro-category, the internal KEGG paths were listed based on the number of sharing model organisms (from the most shared to the less one) and identified by specific numbers following the # sign. The different colors of the hub molecules allow to identify the signaling layer of pertinence. Yellow color indicates upstream molecules, green is TFs, and light blue is the downstream effectors. Created with BioRender.com.

Finally, to give a fuller picture of tenogenesis signaling and its regulatory mechanisms, all paths related to tendon physiology, which showed a high degree of conservation among species, were further considered (see Supplementary Table 10) by showing the relative hubs in Figure 10.

Taking advantage of the enrichment procedure, all paths were populated with hubs belonging to each signaling layer (upstream molecules, TF, and downstream molecules), except for the nervous system paths which did not recognize, to date, controller (hubs) belonging to downstream layers. The upstream layer was the most populated, enclosing the hub nodes of the TGFB, WNT, and mitogenic families. The intermediate layer was represented by specific tendon/skeletal TFs and the downstream layer by nodes involved in ECM deposition activities.

## 4. Discussion

The high prevalence and impact of tendinopathies worldwide involve humans (5–10 cases per 100,000 subjects^9^) and pets or sportive animals^10^ by generating a high clinical demand which cannot be properly managed to date, and, as a result, a progressively increasing trend in expenditure and commitment of healthcare systems is observed.

The present computational study has been carried out to map the molecular information available to date (37 original articles were analyzed) involved in tenogenesis during the embryo–fetal and prepubertal lifetimes (16, 17) to discover which are the main controllers and molecular interactions leading to the early high homeostatic performance. More in detail, this study granted the dissection of tenogenesis molecular events by distinguishing three operative levels and a stage-dependent set of molecules and interactions responsible for signaling differentiation and morphogenesis of tendon tissue. Upstream molecules stimulating the activation of TF responsible for directing the tendon transcriptional program, as well as downstream targets of the above signaling molecules, have been defined. Moreover, this has allowed the identification of new potential interactors belonging to the neuronal and endocrine axes which might be exploited for properly directing signaling events to the definition of a mature tendon structure.

### 4.1. TendonNET nodes and interactions revealed a high degree of preservation in tenogenesis

Based on these premises, the present computational system has been set up to take advantage of the data available in the literature on different model organisms by making species-specific data collections containing molecular interactions, which control tenogenesis in the early life of the most studied organisms. Data collection generated with the literature data allowed us to build seven species-specific networks (TendonNETs) by recognizing the relative nodes and hubs. Their comparative analysis has demonstrated a high level of preservation. Using Venn diagram analysis, several shared clustering nodes were identified; these were primarily represented by paracrine controllers such as IGF, the TGFB family, and neurotrophic factors (see Table 2). Moreover, the analysis of the species-specific TendonNETs allowed us to identify shared hyperconnected nodes (hubs) that represented inside each network the role of controllers of tenogenesis belonging to upstream molecules (TGFB1, TGFB2, TGFB3, and TWSG1) and tendon-related TFs (SCX: see Figure 4). The high degree of preservation of the TendonNETs seems to suggest great feasibility in exploring the molecular machinery controlling tenogenesis encompassing the interspecific barrier also when the animal models are not as closely connected from an evolutionary point of view.

### 4.2. MCL-clustering paths of mice embryo and prepubertal TendonNETs identified common molecular players but stage-specific interactions

The species-specific data collections showed a greater availability of molecular information specifically in the mouse model (mTendonNET) where a sizeable number of the retrieved interactions could split the mTendonNET into two sub-networks: mEmbryo and mPrepubertal TendonNETs. Interestingly, the comparative analysis of the stage-specific TendonNETs revealed that tenogenesis during embryo and prepubertal phase operates using some common nodes which, however, engage strictly stage-dependent interactions occurring at upstream, TF, and downstream levels.

Indeed, the intracellular mediator MAPK1 (otherwise known as ERK2), which was the unique common upstream molecule, works in sensing the signal of mitogenic growth factors in Embryo TendonNETs (IGF and FGF families: see Figure 6), whereas it seems to be preferentially the transducer of the neurotrophic signals (EGR1, NGF, and NGFR) or molecules belonging to the TGFB family (SMAD9 and BMP2) and PI3K during the prepubertal period of life (see Figure 6).

Recent studies appear to confirm a key role for neurotropic axis molecules, such as NGF and its downstream cognates EGR1 and EGR2, in managing the transition from immature tendon cells to tenocytes (41), thus accompanying the structural evolution from neonate to early post-natal tendons (42), which recognize an increased level of complexity.

Additionally, two tendon master TFs, SCX and MKX, were also found to play a crucial role during both embryonic and early post-natal tenogenesis (43) as confirmed by null mice (44–46). Moreover, SCX, under the control of TGF, mediates the transduction signaling of mechanical stimuli throughout the life of the mouse and zebrafish organisms by modulating the spatial regulatory changes of the actin cytoskeleton in stretched tenocytes (47–49).

Similarly, MKX (also known as Iroquois homeobox-like 1) has been recognized as crucial for the regulation of tendon differentiation during embryo development or collagen assembly during post-natal life, particularly in Achilles and tail tendons as confirmed in MKX KO mice (50–52). First, MKX controls the late phase of tendon development (E16.5), when collagen fibrils appear to be normal in terms of size even if they express lower levels of COL1A1, FMOD, DNC, and TNMD (51, 53).

The15ompareson of SCX and MKX KO mice phenotypes suggests that the TFs exert a complementary function, with SCX playing a key role at the beginning of tendon differentiation and MKX in tissue growth and collagen fibril assembly (51).

Furthermore, systems biology revealed that during both the embryonic and early post-natal stages, these shared TFs are involved in exerting indirect control of close districts composing the muscle-skeletal system, albeit through different nodes. More in detail, the two master tendon TFs in mEmbryo TendonNET mainly interacted with MYF5, MYOD, SOX5, SOX6, and PITX1, thus confirming that tendon embryo development requires the coordinated commitments of TFs controlling muscle, cartilage, and bone cell lineages’ commitment.

Even in mPrepubertal TendonNETs, SCX and MKX interact with molecules involved in the maturation of tendon fibrocartilaginous system such as molecules belonging to the Hedgehog (Hh) signaling pathway (GLI1, IHH1, and RUNX2) (54, 55).

Several downstream effectors are represented as common nodes classified into three MCL-clustering-derived paths: collagen fibril formation (COL1A1, COL5A1, and POSTN), ECM organization (THBS4, ELN, ASPN, TNMD, COL1A1, POSTN, TNC, and COL5A1), and keratan sulfate path (PRG4 and FMOD).

The literature evidence confirmed the consistency of the nodes and interaction of mEmbryo TendonNET. More in detail, the early COL1A1 fibril formation path requires POSTNB, COL5A1, and COL1A11A1. Specifically, lowering COL11A1 expression leads to fewer fibril formations *in vivo* and *in vitro* (56–58). At the same time, a synergic action between POSTNB and COL1A1 has been demonstrated as crucial for the regulation of COL1A1 fibrillogenesis leading to final biomechanical properties (4, 59). Furthermore, COL5A1 was found to join these processes during Embryo TendonNET, participating in the network of assembly molecules together with COL11A1 (58, 60–62).

Of note, prepubertal TendonNET highlighted the occurrence of additional interactions which are required to sustain early post-natal fibrillogenesis. Indeed, it appears that COL1A1-primed fibrillogenesis needs to recruit other collagen components including COL3A1 and COL2A1. Although COL3A1 starts to be expressed during embryo development (63), it becomes the major component of the ECM together with COL1A1 in the post-natal stage where it regulates the early events of post-natal tendon development (8, 64). Importantly, a precise ratio of COL3A1 to COL1A1 has been shown to define proper tendon organization, and perturbations have been observed in several pathologies over time (65).

The common nodes of the ECM organization path (THBS4, ELN, ASPN, TNMD, COL1A1, and POSTN) also recognized a stage-specific interaction, clustering with COL11A1, NID2, and TNC in the mEmbryo TendonNET or with COL2A1 and COL3A1, DCN, and LOX in the mPrepubertal one. Focusing on the Embryo TendonNET, POSTNB (66) and COL1A1 (67) activities have been linked to the glycoprotein NID2. NID2 has been shown to interact with COL1A1 in the embryo phase (67) and to be a shared target of SCX together with POSTNB (66).

Consistently, the non-structural matricellular glycoprotein TNC was found as a stage-dependent molecule specifically involved in embryo development and morphogenesis.

On the other hand, THBS4, the glycoprotein regulating many ECM protein–protein and protein–cell interactions (68) interacted with COL2A1 and COL3A1 in the prepubertal TendonNET. Their interaction has consistently been shown to be dependent on the availability of divalent cations Zinc (Zn2+) (69). THBS4 null mice had abnormal collagen fiber size and organization with increased spacing, indicating that THBS4 is required in post-natal life for correct fibril assembly and, most likely, for interaction with other ECM proteins and/or the maintenance of the correct ECM composition in the tendon (68, 70).

Moreover, DCN, the ubiquitous small interstitial dermatan sulfate proteoglycan of mPrepubertal TendonNET, has been recognized to interact with several ECM components including COL1A1, COL6A1, ELN, and THBS (71, 72). DCN was shown to be involved in elastic fiber biology suggesting its involvement in the stabilization of the fibrillin-containing microfibrils and the deposition of tropoelastin onto the microfibrils in the early stages of elastinogenesis (72). Moreover, DCN exerts an anti-adhesive role since it delayed fibroblast attachment on THBS substrate (73).

Scientific studies have also confirmed the interaction of COL1A1, COL2A1, DCN, and ASPN, a proteoglycan expressed in collagen-rich tissues, such as tendon, within mPrepubertal TendonNET (74). More in detail, it has been demonstrated that binding between ASPN and COL1 was inhibited by full-length DCN suggesting a competitive effect between ASPN and DCN in binding to collagen (74).

Finally, the Keratan Sulfate Biosynthesis path included FMOD and PRG4 as stage-independent nodes which cluster with CHST5 and KERA within the mEmbryo TendonNET and with LUM in the mPrepubertal one.

Limited information is available on the Embryo TendonNET KERA node, a proteoglycan previously thought to be ‘corneal-specific’ and currently recognized as abundant also in the tendon where it regulates collagenous ECM (75). Mapping the embryo development, KERA was found to interact with lubricin, also known as PRG4 or superficial zone protein, a boundary lubricant anti-adhesive component in diverse tissues by protecting them against frictional forces (76).

Conversely, LUM became a preferential interactor within the Prepubertal TendonNET. Consistently, either *in vitro* and *in vivo* data confirmed that LUM and FMOD share the same binding region on COL1A1, hence affecting collagen fibrillogenesis (77–79).

In contrast to the MCL-clustering analysis, direct bibliographical source analysis appears to indicate a stage-independent role for LUM. Although evidence is limited, some research findings indicate that LUM is involved in fibrillogenesis during tendon organogenesis, with decreased LUM having an inverse behavior with respect to FMOD, implying that the different observed patterns might be important in regulating this process (80). The complementary stage-dependent phenotype of tendons derived from LUM and FMOD KO strongly supported this hypothesis (80).

### 4.3. Research evidence collected from KO models embodies the consistency of node interactions of the enriched TendonNETs

Methodologically, string enrichment was adopted to further fill out the molecular information flow. This computational approach allowed us to significantly increase the number of nodes within each species-specific network at the three operative layers of control, by identifying new upstream node molecules belonging to TGF, WNT, mitogenic, hormonal, and metabolic axes (see Figure 8). Among them, TGFB1, TGFB2, TGFB3, BMP7, and TWSG1 assumed a conserved role of controllers (hubs) of tenogenesis as demonstrated by the high degree of interconnection in m, h, e, b, and g Enriched TendonNETs.

Accordingly, the central role of TGFB in tendon development was corroborated by several pieces of evidence displaying the expression of TGFB ligands in tendon progenitor cells and, more importantly, the failure of tendon development after TGFBR2 or TGFB2 genetic deletions (81–83). Also, SMAD3 defective mice, a TGFB intracellular signaling member, led to altered tendon architecture (84).

WNT cascade was also enriched by the String approach. Interestingly, a recent study by Kishimoto and colleagues gave some insights into the contribution of the WNT cascade on tenogenesis. Indeed, the authors showed that WNT antagonizes the TGFB path and that a perturbation of their crosstalk might be responsible for tendon healing conditions (85).

Growth factors of the FGF family linking for the mitogenic cascades involving the intracellular mediators belonging to the MAPK family were also enriched, suggesting, according to preliminary literature data on null mice, a regulatory role of tenogenesis for mitogenic signal events (86–88).

Furthermore, enrichment in nodes belonging to the hormonal cascade, such as FST and NR4A1, allowed us to take into consideration additional mechanisms working concomitantly to tightly regulate the differentiation signal of the above-mentioned cascades. For example, the role of the FST hormone may unfold the master blocking signal of TGFB-mediated tenogenesis (64).

Moreover, the hormone-retinoid receptor NR4A1, a new node in Enriched TendonNET, was found to regulate the expression of the tenogenic differentiation genes SCX and TNMD in rat tendon-derived stem cells and, most importantly, its activity was enhanced by photo-biomodulation therapy (89), a useful approach under investigation for the development of clinical therapeutic protocols enhancing tissue regeneration (90).

Additionally, String enrichment valorized the tendon influence on metabolic axis members. This computational evidence emphasizes an auxiliary role of the metabolism in controlling tendon homeostasis during development. One of the key members of the metabolic axis enriched by the present study was HIF1. Even though few studies focused on HIF action during tendon development, a murine phenotype corroborating its involvement in events related to tenogenesis has been observed in HIF defective mice which showed short limbs (ID of the KO mouse available in the^11^ with the ID: MGI:3621466).

Consistent studies have been collected focusing on its role during tendon regeneration. Importantly, HIF1 has been described to be implicated in managing glycolytic reprogramming following a stress stimulus in a *in vivo* mouse model of tendinopathy (91). Furthermore, HIF1 increased activity was found in biopsies of patients with tendinopathies (92). Accordingly, its inhibition has been proposed as a solution to attenuate Achilles tendinopathies (93).

Moving to the TF layer, SCX and MKX displayed a conserved hub role into m, e, g, d, and rTendonNETs.

The master role of the tendon controller of SCX is recognized in controlling diverse target genes: 32 activated by the TF and 17 suppressed during early tendon development (66). Among the target genes EYA1, SIX2, MKX, TNMD, FMOD, and IGF1, SPARC has been retrieved by the present computational analysis.

Second, SCX upstream molecules, such as those of the TGFB and FGF cascades, which coordinately induce the development of axial and limb tendon progenitors via SCX action, have been recognized (48, 81–83, 94–97).

Differently, the upstream MKX expression has not been clarified yet, but several downstream molecules identified by the NETs have been confirmed by null mice models (51).

Like SCX, MKX was found to be involved in several ECM molecules during the peri e post-natal lifetime. MKX appeared to regulate COL11A1 by influencing the pre-natal expression and controlling the later process of deposition (post-natal) (51, 53). Collagen fibril assembly was also found to be inhibited through the downregulation of tendon-associated molecules binding to fibril surfaces such as DCN, FMOD, and LUM (51, 53).

Because proper tendon development is the result of a coordinated commitment of the other skeletal muscle cell lineages (muscle, cartilage, and tendon) (98), additional TF, ensuring mutual control, was consistently retrieved using the computational approach to ensure mutual control. Among them, MEOX belonged to dTendonNET, and SIX2, EYA, and FHL2 derived from gTenonNETs. MYOD1 and FOXH1 were retrieved by String enrichment in bStringNET. Of note, FHL2 and SIX2 were hubs of the gTendonNET.

Neuronal-related TF were finally included and represented the mediators of the NGF/NGFR signaling that was recognized upstream in the mTendonNET. Neuronal-related TF included ERG1, working as a hub in rTendonNET and TCF7L2 and retrieved as a node of the gEnriched TendonNET.

Analogously, String enrichment fills out the downstream layer by adding new nodes and hubs responsible for ECM deposition, assembly, maturation, and binding. Among these, some members of the ECM deposition family including COL1A1 and ACAN, and ECM assembly nodes, such as BGN, were identified as hubs in m and bTendonNETs, respectively.

Consistent with the signaling flow, two molecules retrieved by the present analysis, COL1A1 and TNMD, were found to be direct downstream targets of the SCX activity.

Also, the promoter of COL1A1 was found to be responsive to additional TF, such as ERG1 and ERG2, which have been found to be recruited to the COL1A1 promoter region and promote its transactivation (99).

Like COL1A1, other ECM molecules of the Enriched TendonNETs, such as COL1A2, COL3A1, and COL5A1, have been described as targets of EGR1 and EGR2 during tendon cell differentiation in mouse and chick limbs (99).

A key role for ECM organization was also recognized for COL6A1, a new node of the dEnriched TendonNET. Literature data corroborate its inclusion into the network of events supporting tendon development. Its peculiar function has been associated with the fibroblast-matrix interface. Indeed, COL6A1 was found enriched in peri-cellular regions of tendon fibroblasts. However, the precise mechanism and interaction in which it is involved still need to be defined (100).

The class of molecules responsible for ECM assembly was densely filled by String enrichment with new members, such as SPARC, LUM, and POSTNB. Although data concerning molecular mechanisms encompassing their influence on tendon development are still required, defective mouse models supported their function in tenogenesis (77, 80, 101–105).

### 4.4. Computational enrichment of node interactions of early tenogenesis empowers four physiological pathways, including the endocrine and nervous systems

Finally, to define the physiological processes in which the Enriched TendonNETs nodes participate, a KEGG analysis was performed, which identified four macro-categories of physiological paths including (I) growth, differentiation, and survival, (II) morphogenesis and cell motility, and (III) endocrine and (IV) nervous systems.

Of note, the nervous and endocrine systems, which populated almost all NETs following enrichment, emerged as crucial paths for managing early tenogenesis (see Table 5).

In more detail, the nervous system included paths related to neurotrophin signaling and axon guidance recognized as key mediators of neuronal factors such as NGF, NGFR (otherwise known as p75NTR), EGR, TCF7L2, NAB1, and NAB2 nodes.

NGF and its receptors were found in Achilles tenocytes (106), and *in vivo* rats studies confirmed that NGF signaling is associated either with connective tissue homeostasis or pathological tendon characterized by adverse collagen deposition. NGF signaling, for example, is compromised during tendon healing in diabetic organisms (107) where the accumulation of advanced glycation end-products (AGE) mediated changes in ECM collagen by primarily damaging the process of collagen fibrils’ reorganization (108).

The action of NGF signaling is switched on through nerve-related receptor NGFR and Tyrosine receptor kinases (Trk-a and Trk-b). Of note, the latter receptor family can be activated also by other growth factors regulating cell proliferation and differentiation whose activity might be useful to reinforce the signaling cascade promoted by NGF (109).

In line with this premise, the topological analysis of Tendon EnrichedNETs unveiled as a controller node (hub) within the nervous system, and the mitogenic intracellular mediator MAPK1 and the morphogenic growth factor BMP7 (see Figure 10) were involved in this process of indirect paths activation of NGF signaling.

NGF signaling, on the other hand, has been shown to activate ERG1, one of the neuronal TF identified by Tendon NETs, by activating downstream key tendon-related genes that code for molecules involved in ECM deposition (COL1, COL3, COL5, COL6, and COL14), assembly (BGN and DCN), and maturation (FN1) (110).

In addition to ERG1, another neurogenic TF, TCF7L2, has been identified. It was found to be downstream of the WNT cascade in various contexts and to be temporally regulated to ensure the smooth development of oligodendroglia lineage cells (111, 112) of the brain (113, 114), thus potentially playing a role in numerous cellular processes ranging from early development to adult tissue homeostasis (115–117).

Notably, two neuronal targets populated the downstream layer in the rEnriched TendonNETs, namely, Nab1 and Nab2. Nab1 and Nab2 are neural tissue-specific actin filament-binding proteins involved in the dynamic organization of actin filaments controlling morphology, migration, and function during neurite formation and axon outgrowths that have never been studied until now in tendon systems.

Even though Nab1 and Nab2 showed closely related aminoacidic sequences, they display distinct functions, modulatory phosphorylation/dephosphorylation patterns, and subcellular localization (118). More in detail, Nab1 is present in spines and the lamellipodia of the growth cone during the development of neurons (119), whereas Nab2 is also named Spinophilin for its exclusive localization in dendritic spines (120). Nab1 operates as a neuronal-specific scaffold protein that binds at the same time F-actin and diverse functional proteins were implicated in cytoskeletal dynamics transduction responses [e.g., phosphatase 1 (PP1) and p70 ribosomal S6 protein kinase]. Taking advantage of these multiprotein complexes, Nab1 controls actin rearrangement and membrane activity involved in the promotion of dendritic spine formation and function of mammalian neurons (121–123).

Analogously, Nab2 acts as a scaffold, anchoring more than 30 partner molecules including cytoskeletal factors, cell adhesion molecules, guanine nucleotide exchange factors (GEF), a regulator of G-protein signaling protein, membrane receptors, and ion channels (118). Although the physiological significance of the majority of these interactions is unknown, scientific evidence to date indicates that Nab2 plays an important role in the regulation of spine morphology and density, synaptic plasticity, and neuronal migration, thus contributing to nervous system development and remodeling specifically inducing the regenerating growth cones to repair the functional neuronal connections, at least, in the central nervous system after injury (118). However, there is no scientific evidence to date that Nab1 and Nab2 are involved in the mechanisms of tendon tissue differentiation, maturation, and homeostasis control.

The endocrine pathway is also noteworthy, as its influence on tendon morphology and function was only recently discovered. More evidence has been gathered to date to demonstrate a negative relationship between tendon homeostasis and endocrine disorders. Indeed, *in vitro* and *in vivo* studies on animals and humans have revealed that subjects with hormonal imbalances have changes in their tendon microarchitecture and function that are solely hormone-dependent (124).

In more detail, the endocrine system was composed of four main pathways including relaxin, aldosterone regulating sodium adsorption, prolactin, and melanogenesis signaling.

The available scientific data confirmed that metabolic changes caused by relaxin and prolactin hormonal disorders could induce tendinopathies affecting both tendon and ligament structures during pregnancy (125). Specifically, relaxin has been found to alter the properties of cartilage and tendons by activating collagenase enzymes (126).

Similarly, increased systemic prolactin concentrations during pregnancy have been linked to the release of proinflammatory cytokines and matrix metalloproteinases responsible for connective tissue disease from fibroblast-like cells. The observation of decreased tendon reflexes of the patellar and Achilles tendons in patients with high serum prolactin levels supported this hypothesis (127, 128).

Furthermore, aldosterone signaling has been shown to influence muscle skeletal metabolism by serving as a potential etiological cause of chronic disorders in which aldosterone overproduction induces a variety of muscle conditions (129) or tendon degeneration (130, 131). As proof, a case report study showed a correlation between the aldosteronism condition and calcification of the Achilles tendons and knees (130). Accordingly, Saiki and colleagues showed impaired tendon reflexes (131).

In contrast, there is no evidence of a link between the path of melanogenesis and tendon physiology or pathology. Although many efforts have been made to clarify the relationship between the nervous and endocrine axis and changes in the architecture and function of the Achilles tendon, the topic still warrants further investigation.

### 4.5. Conclusion

Overall, the data show that computational approaches were able to design tight and time-dependent molecular interactions, either empowering each signaling layer or establishing how information flow moves within the network, converging toward the more connected nodes (hub) of TendonNET, the main controller of early tenogenesis.

First, the high degree of preservation of the Tendon and Enriched TendonNETs seems to suggest great feasibility in navigating the molecular interaction controlling tenogenesis encompassing the interspecific barriers, even when the animal models are not as closely related evolutionarily.

Second, the computational map of tenogenesis established a stepwise process recognizing stage-specific molecular interactions that merit further experimental investigation to improve biological knowledge of this system as a prerequisite for developing the targeted therapeutic strategies that the medical and veterinary sectors have been waiting for. Given the variety of molecular interconnections revealed by the embryonic and prepubertal mouse TendonNETs, it seems reasonable to favor the latter as the experimental model with the greatest translation value for the discovery of mechanisms that could be used in adult tissue.

Finally, the present research unveiled a hierarchical organization of the signals managing tendon homeostasis during development identifying, after the computational enrichment, a new and not fully characterized level of signals belonging to the nervous and endocrine systems. Molecules identified with their positive influence during normal tendon development even in an early post-natal lifetime could be exploited for confirming experimentally its role in controlling tissue homeostasis and healing during adulthood.

## Data availability statement

The original contributions presented in the study are included in the article/Supplementary material, further inquiries can be directed to the corresponding author.

## Author contributions

AP and BB contributed to the design of the study, drafted the study, and revised critically the upstream signaling molecules and transcription factors results. AC-V, AM-H, MF, and MC generated data collection. NB made computational analyses. VR, MEK, and GP contributed to the draft of the work revising critically downstream molecules results. AM-H and PB contributed to the supervision of the manuscript, revision, coordination, and fund recovery. All authors contributed to the article and approved the submitted version.

## Funding

This research was funded by the European Union—Next Generation EU. Project Code: ECS00000041; Project CUP: C43C22000380007; Project Title: Innovation, digitalization, and sustainability for the diffused economy in Central Italy—VITALITY. AC-V, AM-H, and MF were supported by the doctorate project “Perspective for Future Innovation in Tendon Repair H2020-MSCA-ITN-EJD-P4 FIT” (Grant Agreement ID: 955685).

## Conflict of interest

The authors declare that the research was conducted in the absence of any commercial or financial relationships that could be construed as a potential conflict of interest.

## Publisher’s note

All claims expressed in this article are solely those of the authors and do not necessarily represent those of their affiliated organizations, or those of the publisher, the editors and the reviewers. Any product that may be evaluated in this article, or claim that may be made by its manufacturer, is not guaranteed or endorsed by the publisher.

## Figures and Tables

**Table:** 

ACAN	AGGRECAN
ACTA1	ACTIN ALPHA 1, SKELETAL MUSCLE
ACVR1	ACTIVIN RECEPTOR 1
ACVR1B	ACTIVIN RECEPTOR 1
ACVR1BLIKE	ACVR1B ACTIVIN A RECEPTOR TYPE 1B
ACVR1C	ACTIVIN RECEPTOR 1C
ACVR2A	ACTIVIN RECEPTOR 2A
ACVR2B	ACTIVIN RECEPTOR 2B
ACVRL1	ACTIVIN A RECEPTOR LIKE TYPE 1
ADGRB3	ADHESION G-PROTEIN-COUPLED RECEPTOR B3
ADGRG2	ADHESION G-PROTEIN-COUPLED RECEPTOR G2
AFF3	ALF TRANSCRIPTION ELONGATION FACTOR 3
AK1	ADENYLATE KINASE 1
ALPL	ALKALINE PHOSPHATASE, BIOMINERALIZATION ASSOCIATED
AMPK	AMP-ACTIVATED CATALYTIC SUBUNIT ALPHA 1 PROTEIN KINASE
APC	APC REGULATOR OF WNT SIGNALING PATHWAY
AQP1	AQUAPORIN 1
ASPN	ASPORIN
ATF3	ACTIVATING TRANSCRIPTION FACTOR 3
BCL9	BCL9 TRANSCRIPTION COACTIVATOR
BGN	BIGLYCAN
BMP2	BONE MORPHOGENETIC PROTEIN 2
BMP4	BONE MORPHOGENETIC PROTEIN 4
BMP5	BONE MORPHOGENETIC PROTEIN 5
BMP6	BONE MORPHOGENETIC PROTEIN 6
BMP7	BONE MORPHOGENETIC PROTEIN 7
BMPER	BMP BINDING ENDOTHELIAL REGULATOR
BMPR1	BONE MORPHOGENETIC PROTEIN RECEPTOR TYPE 1
BMPR1A	BONE MORPHOGENETIC PROTEIN RECEPTOR TYPE 1A
BMPR1AA	BONE MORPHOGENETIC PROTEIN RECEPTOR TYPE 1AA
BMPR1AB	BONE MORPHOGENETIC PROTEIN RECEPTOR TYPE 1AB
BMPR1BA	BONE MORPHOGENETIC PROTEIN RECEPTOR TYPE 1BA
BMPR2	BONE MORPHOGENETIC PROTEIN RECEPTOR TYPE 2
C1QTNF3	C1Q AND TNF RELATED 3
CACNB2	CALCIUM VOLTAGE-GATED CHANNEL AUXILIARY SUBUNIT BETA 2
CBFB	CORE-BINDING FACTOR SUBUNIT BETA
CCBE1	COLLAGEN AND CALCIUM BINDING EGF DOMAINS 1
CCDC85A	COILED-COIL DOMAIN CONTAINING 85A
CCDC88A	COILED-COIL DOMAIN CONTAINING 88A
CCN1	CELLULAR COMMUNICATION NETWORK FACTOR 1
CDK1	CYCLIN-DEPENDENT KINASE 1
CHDL1	CHROMODOMAIN HELICASE DNA BINDING PROTEIN 1 LIKE
CHDL2	CHROMODOMAIN HELICASE DNA BINDING PROTEIN 2 LIKE
CHRD	CHORDIN
CHST5	CARBOHYDRATE SULFOTRANSFERASE 5
CLEC3B	C-TYPE LECTIN DOMAIN FAMILY 3 MEMBER B
CLIP2	CAP-GLY DOMAIN CONTAINING LINKER PROTEIN 2
COL11A1	COLLAGEN TYPE XI ALPHA 1 CHAIN
COL14A1	COLLAGEN TYPE XIV ALPHA 1 CHAIN -
COL1A1	COLLAGEN TYPE I ALPHA 1 CHAIN
COL22A1	COLLAGEN TYPE XXII ALPHA 2 CHAIN
COL2A1	COLLAGEN TYPE II ALPHA 1 CHAIN
COL2A2	COLLAGEN TYPE II ALPHA 2 CHAIN
COL3A1	COLLAGEN TYPE III ALPHA 1 CHAIN
COL4A	COLLAGEN TYPE IV ALPHA 4 CHAIN
COL5A1	COLLAGEN TYPE V ALPHA 1 CHAIN
COL5A2	COLLAGEN TYPE V ALPHA 2 CHAIN
COL6A1	COLLAGEN TYPE VI ALPHA 1 CHAIN
COMP	CARTILAGE OLIGOMERIC MATRIX PROTEIN
CTNNA1	CATENIN ALPHA 1
CTNNB1	CATENIN BETA 1
CTNNBIP1	CATENIN BETA INTERACTING PROTEIN 1
CX32	GAP JUNCTION PROTEIN BETA 1
CX43	GAP JUNCTION PROTEIN ALPHA 1
CXCL12	C-X-C MOTIF CHEMOKINE LIGAND 12
CYR61	CELLULAR COMMUNICATION NETWORK FACTOR 1
DAN	NBL1, DAN FAMILY BMP ANTAGONIST
DCN	DECORIN
DIO2	IODOTHYRONINE DEIODINASE 2
DOCK4	DEDICATOR OF CYTOKINESIS 4
DSC2	DESMOCOLLIN 2
DUSP6	DUAL SPECIFICITY PHOSPHATASE 6
E2F4	E2F TRANSCRIPTION FACTOR 4
EGR1	EARLY GROWTH RESPONSE 1
ELN	ELASTIN
ENG	ENDOGLIN
ENPP2	ECTONUCLEOTIDE PYROPHOSPHATASE/PHOSPHODIESTERASE 2
EP300	E1A BINDING PROTEIN P300
EPHA3	EPH RECEPTOR A3
EPHA4	EPH RECEPTOR A4
ERK2	MITOGEN-ACTIVATED PROTEIN KINASE 1
EYA1	EYA TRANSCRIPTIONAL COACTIVATOR AND PHOSPHATASE 1
FAT3	FAT ATYPICAL CADHERIN 3
FGF10	FIBROBLAST GROWTH FACTOR 10
FGF2	FIBROBLAST GROWTH FACTOR 2
FGF23	FIBROBLAST GROWTH FACTOR 23
FGF4	FIBROBLAST GROWTH FACTOR 4
FGF6	FIBROBLAST GROWTH FACTOR 6
FGF8	FIBROBLAST GROWTH FACTOR 8
FGFR1	FIBROBLAST GROWTH FACTOR RECEPTOR 1
FHL2	FOUR AND A HALF LIM DOMAINS 2
FIBIN	FIN BUD INITIATION FACTOR HOMOLOG
FKBP1	FKBP PROLYL ISOMERASE 1
FKBP1A	FKBP PROLYL ISOMERASE 1A
FMOD	FIBROMODULIN
FN1	FIBRONECTIN 1
FOS	FOS PROTO-ONCOGENE, AP-1 TRANSCRIPTION FACTOR SUBUNIT
FOSB1	FOSB PROTO-ONCOGENE, AP-1 TRANSCRIPTION FACTOR SUBUNIT
FOSL1	FOS LIKE 1, AP-1 TRANSCRIPTION FACTOR SUBUNIT
FOXH1	FORKHEAD BOX H1
FST	FOLLISTATIN
FSTL1	FOLLISTATIN LIKE 1
FSTL5	FOLLISTATIN LIKE 5
GDF5	GROWTH DIFFERENTIATION FACTOR 5
GDF6	GROWTH DIFFERENTIATION FACTOR 6
GDF6A	GROWTH DIFFERENTIATION FACTOR 6A
GLI1	GLI FAMILY ZINC FINGER 1
GPC3	GLYPICAN 3
GSK3B	GLYCOGEN SYNTHASE KINASE 3 BETA
HDAC4	HISTONE DEACETYLASE 4
HIF1A	HYPOXIA INDUCIBLE FACTOR 1 SUBUNIT ALPHA
HOXA13	HOMEOBOX A13
HOXC10	HOMEOBOX C10
HSPG2	HEPARAN SULFATE PROTEOGLYCAN 2
HTRA3	HTRA SERINE PEPTIDASE 3
IBSP	INTEGRIN BINDING SIALOPROTEIN
IGF1	INSULIN GROWTH FACTOR 1
IGF1-LIKE	INSULIN LIKE GROWTH FACTOR 1
IGF2BP1	INSULIN LIKE GROWTH FACTOR 2 MRNA BINDING PROTEIN 1
IGH3	TRANSFORMING GROWTH FACTOR BETA INDUCED
IHH1	INDIAN HEDGEHOG SIGNALING MOLECULE 1
INHBA	INHIBIN SUBUNIT BETA A
ITGA1	INTEGRIN SUBUNIT ALPHA 1
ITGA5	INTEGRIN SUBUNIT ALPHA 5
ITGB1	INTEGRIN SUBUNIT BETA 1
ITGB2	INTEGRIN SUBUNIT BETA 2
ITGB3	INTEGRIN SUBUNIT BETA 3
ITGB6	INTEGRIN SUBUNIT BETA 6
ITGB8	INTEGRIN SUBUNIT BETA 8
JUN B	JUN PROTO-ONCOGENE, AP-1 TRANSCRIPTION FACTOR SUBUNIT B
JUN D	JUN PROTO-ONCOGENE, AP-1 TRANSCRIPTION FACTOR SUBUNIT D
KERA	KERATOCAN
KLB	KLOTHO BETA
LDB2	LIM DOMAIN BINDING 2
LEF1	LYMPHOID ENHANCER BINDING FACTOR 1
LINGO2	LEUCINE-RICH REPEAT AND IG DOMAIN CONTAINING 2
LOX	LYSYL OXIDASE
LTPB1	LATENT TRANSFORMING GROWTH FACTOR BETA BINDING PROTEIN 1
LUM	LUMICAN
MAP K1	MITOGEN-ACTIVATED PROTEIN KINASE 1
MAP K3	MITOGEN-ACTIVATED PROTEIN KINASE 3
MAP K8	MITOGEN-ACTIVATED PROTEIN KINASE 8
MAP K9	MITOGEN-ACTIVATED PROTEIN KINASE 9
MEF2C	MYOCYTE ENHANCER FACTOR 2C
MEOX1	MESENCHYME HOMEOBOX 1
MKX	MOHAWK HOMEOBOX
MKXA	MOHAWK HOMEOBOX A
MKXB	MOHAWK HOMEOBOX
MMP1	MATRIX METALLOPEPTIDASE 1
MMP11	MATRIX METALLOPEPTIDASE 11
MMP13	MATRIX METALLOPEPTIDASE 13
MMP16	MATRIX METALLOPEPTIDASE 16
MMP2	MATRIX METALLOPEPTIDASE 2
MMP3	MATRIX METALLOPEPTIDASE 3
MSN	MOESIN
MTCL1	MICROTUBULE CROSSLINKING FACTOR 1
MTCL1	MICROTUBULE CROSSLINKING FACTOR 1
MYF5	MYOGENIC FACTOR 5
MYOD1	MYOGENIC DIFFERENTIATION 1
NAALAD2	N-ACETYLATED ALPHA-LINKED ACIDIC DIPEPTIDASE 2
NAALAD2	N-ACETYLATED ALPHA-LINKED ACIDIC DIPEPTIDASE 2
NAB1	NGFI-A BINDING PROTEIN 1
NAB2	NGFI-A BINDING PROTEIN 2
NEDD4L	NEDD4 LIKE E3 UBIQUITIN PROTEIN LIGASE
NGF	NERVE GROWTH FACTOR
NGFR	NERVE GROWTH FACTOR RECEPTOR
NID2	NIDOGEN 2
NOG	NOGGIN
NR4A1	NUCLEAR RECEPTOR SUBFAMILY 4 GROUP A MEMBER 1
OLFML2B	OLFACTOMEDIN LIKE 2B
PCOLCE	PROCOLLAGEN C-ENDOPEPTIDASE ENHANCER
PI3K	PHOSPHATIDYLINOSITOL-4,5-BISPHOSPHATE 3-KINASE
PITX1	PAIRED LIKE HOMEODOMAIN 1
PLCG1	PHOSPHOLIPASE C GAMMA 1
PLCH1	PHOSPHOLIPASE C ETA 1
POSTN	PERIOSTIN
POSTNB	PERIOSTIN B
PRG4	PROTEOGLYCAN 4
RAVER2	RIBONUCLEOPROTEIN, PTB BINDING 2
RCN3	RETICULOCALBIN 3
RFLNB	REFILIN B
RNASE4	RIBONUCLEASE A FAMILY MEMBER 4
RUNX2	RUNX FAMILY TRANSCRIPTION FACTOR 2
SCUBE2	SIGNAL PEPTIDE, CUB DOMAIN, AND EGF-LIKE DOMAIN CONTAINING 2
SCX	SCLERAXIS BHLH TRANSCRIPTION FACTOR
SCXA	SCLERAXIS BHLH TRANSCRIPTION FACTOR
SDC4	SYNDECAN 4
SFRP2	SECRETED FRIZZLED-RELATED PROTEIN 2
SHC1	SHC ADAPTOR PROTEIN 1
SHP2	PROTEIN TYROSINE PHOSPHATASE NON-RECEPTOR TYPE 11
SIX1	SIX HOMEOBOX 1
SIX2	SIX HOMEOBOX 2
SKIA	SKI PROTO-ONCOGENE
SKILA	SKI-LIKE PROTO-ONCOGENE
SMAD1	SMAD FAMILY MEMBER 1
SMAD2	SMAD FAMILY MEMBER 2
SMAD3	SMAD FAMILY MEMBER 3
SMAD3A	SMAD FAMILY MEMBER 3A
SMAD3B	SMAD FAMILY MEMBER 3B
SMAD4	SMAD FAMILY MEMBER 4
SMAD5	SMAD FAMILY MEMBER 5
SMAD7	SMAD FAMILY MEMBER 7
SMAD8	SMAD FAMILY MEMBER 8
SMO	SMOOTHENED, FRIZZLED CLASS RECEPTOR
SMOC2	SPARC-RELATED MODULAR CALCIUM BINDING 2
SMURF2	SMAD-SPECIFIC E3 UBIQUITIN PROTEIN LIGASE 2
SOX5	SRY-BOX TRANSCRIPTION FACTOR 5
SOX6	SRY-BOX TRANSCRIPTION FACTOR 6
SOX9	SRY-BOX TRANSCRIPTION FACTOR 9
SP1	SP1
SP7	SP7 TRANSCRIPTION FACTOR
SPARC	SECRETED PROTEIN ACIDIC AND CYSTEINE RICH
SPP1	SECRETED PHOSPHOPROTEIN 1
SSC5D	SCAVENGER RECEPTOR CYSTEINE RICH FAMILY MEMBER WITH 5 DOMAINS
ST8SIA1	ST8 ALPHA-N-ACETYL-NEURAMINIDE ALPHA-2,8-SIALYLTRANSFERASE 1
SULF1	SULFATASE 1
SYT7	SYNAPTOTAGMIN 7
TBX4	T-BOX TRANSCRIPTION FACTOR 4
TCF7L2	TRANSCRIPTION FACTOR 7 LIKE 2
TGFB1	TRANSFORMING GROWTH FACTOR BETA 1
TGFB2	TRANSFORMING GROWTH FACTOR BETA 2
TGFB3	TRANSFORMING GROWTH FACTOR BETA 3
TGFBLIKE	TRANSFORMING GROWTH FACTOR BETA-LIKE
TGFBR1	TRANSFORMING GROWTH FACTOR BETA RECEPTOR 1
TGFBR1A	TRANSFORMING GROWTH FACTOR BETA RECEPTOR 1A
TGFBR1B	TRANSFORMING GROWTH FACTOR BETA RECEPTOR 1B
TGFBR2	TRANSFORMING GROWTH FACTOR BETA RECEPTOR 2
TGFBR3	TRANSFORMING GROWTH FACTOR BETA RECEPTOR 3
TGIF1	TGFB INDUCED FACTOR HOMEOBOX 1
THBS2	THROMBOSPONDIN 2
THBS4	THROMBOSPONDIN 4
TIMP2	TIMP METALLOPEPTIDASE INHIBITOR 2
TLL1	TOLLOID LIKE 1
TLR2	TOLL-LIKE RECEPTOR 2
TMEM44	TRANSMEMBRANE PROTEIN 44
TNC	TENASCIN C
TNFSF11	TNF SUPERFAMILY MEMBER 11
TNMD	TENOMODULIN
TP53	TUMOR PROTEIN P53
TRIM33	TRIPARTITE MOTIF CONTAINING 33
TWIST	TWIST FAMILY BHLH TRANSCRIPTION FACTOR 1
TWSG1	TWISTED GASTRULATION BMP SIGNALING MODULATOR 1
WNT3	WNT FAMILY MEMBER 3
WNT3A	WNT FAMILY MEMBER 3A
WNT6	WNT FAMILY MEMBER 6
WNT7	WNT FAMILY MEMBER 7
XIRP2A	XIN ACTIN BINDING REPEAT CONTAINING 2
ZFP185	ZINC FINGER PROTEIN 185 WITH LIM DOMAIN
ZFYVE9	ZINC FINGER FYVE-TYPE CONTAINING 9

## Footnotes

^1^ http://www.prisma-statement.org/

^2^ https://apps.webofknowledge.com/WOS_AdvancedSearch

^3^ http://www.cytoscape.org

^4^ https://string-db.org/

^5^ http://www.genome.jp/kegg/

^6^ https://bioinformatics.psb.ugent.be/webtools/Venn/

^7^ http://micans.org/mcl/

^8^ www.genome.jp

^9^ https://www.grandviewresearch.com/industry-analysis/tendonitis-treatment-market-report

^10^ https://www.grandviewresearch.com/industry-analysis/veterinary-orthopedics-market

^11^ https://www.informatics.jax.org
