# Supplementary material for: Mammal comparative tendon biology: advances in regulatory mechanisms through a computational modeling
Source: Front Vet Sci. 2023 Apr 27;10:1175346. doi: 10.3389/fvets.2023.1175346 (PMC10174257; doi:10.3389/fvets.2023.1175346)
Supplement: Supplementary file 7 [file Data_Sheet_7.pdf]

*Supplementary File 7*

# Mammal comparative tendon biology: advances in regulatory mechanisms through a computational modelling

**Alessia Peserico[1#*], Barbara Barboni[1#], Valentina Russo[1], Nicola Bernabò[1], Mohammad El Kathib[1], Giuseppe Prencipe[1], Adrián Cerveró-Varona[1], Arlette Alina Haidar Montes[1], Melisa Faydaver[1], Maria Rita Citeroni[1], Paolo Berardinelli[1], Annunziata Mauro[1].**

[1]Unit of Basic and Applied Sciences, Department of Bioscience and Technology for Food, Agriculture and Environment, University Teramo, Via R. Balzarini 1, 64100 Teramo, Italy

**# These authors equally contributed to this work**

*** Correspondence:**
Alessia Peserico
apeserico@unite.it

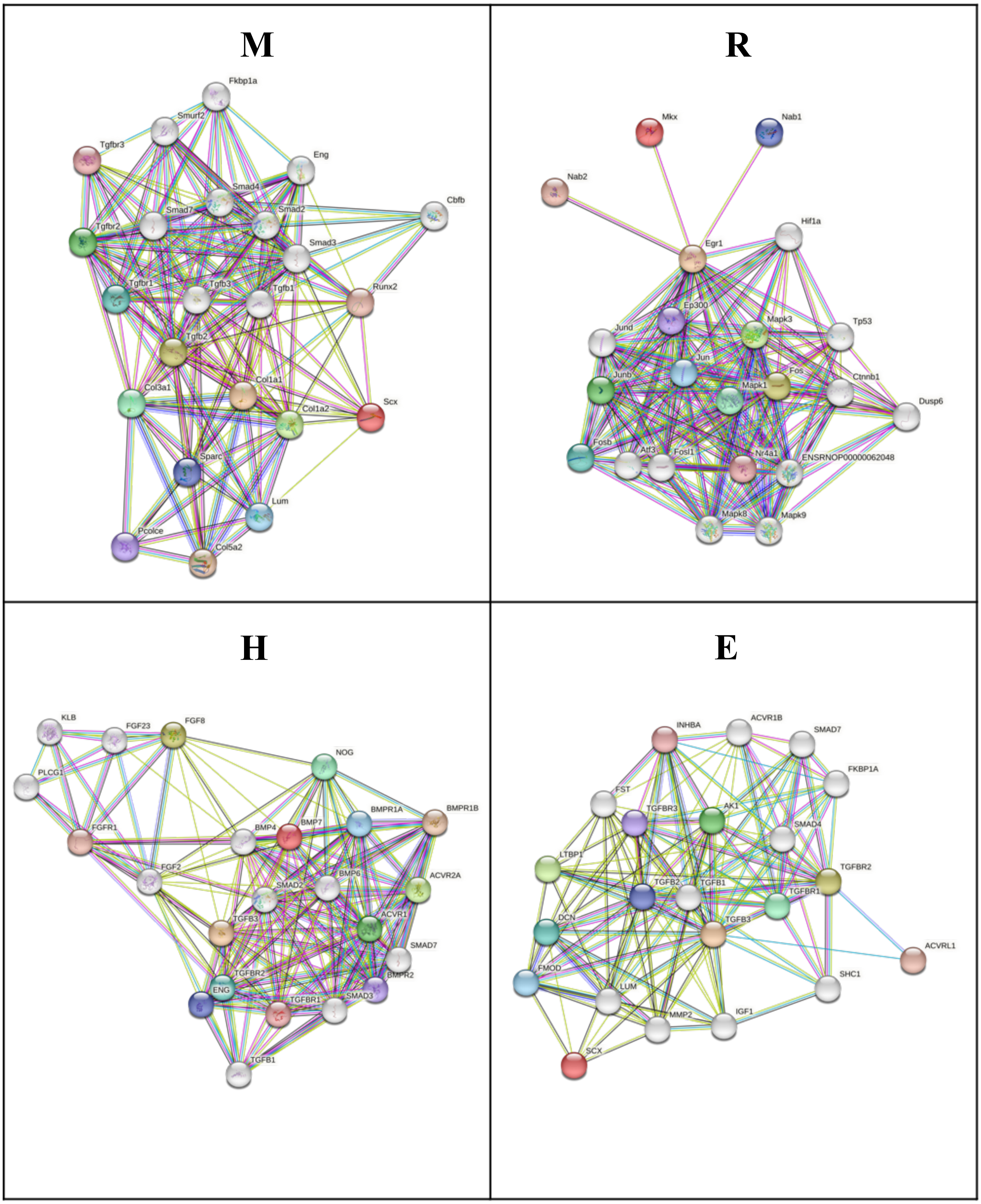
M
Fkbp1a
Smurf2
Tgfbr3
Eng
Smad4
Smad7
Smad2
Cbfb
Tgfbr2
Smad3
Tgfbr1
Tgfb3
Tgfb1
Runx2
Tgfb2
Col1a1
Col3a1
Col1a2
Scx
Sparc
Lum
Pcolce
Col5a2
R
Mkx
Nab1
Nab2
Hif1a
Egr1
Ep300
Mapk3
Tp53
Jund
Jun
Junb
Fos
Ctnnb1
Mapk1
Dusp6
Fosb
Atf3
Fosl1
Nr4a1
ENSRNOP00000062048
Mapk8
Mapk9
H
KLB
FGF23
FGF8
NOG
PLCG1
BMPR1B
BMPR1A
FGFR1
BMP4
BMP7
FGF2
BMP6
ACVR2A
SMAD2
TGFB3
ACVR1
SMAD7
TGFBR2
BMPR2
ENG
TGFBR1
SMAD3
TGFB1
E
INHBA
ACVR1B
SMAD7
FKBP1A
FST
TGFBR3
AK1
SMAD4
LTBP1
TGFBR2
TGFB2
TGFB1
TGFBR1
DCN
TGFB3
ACVRL1
FMOD
SHC1
LUM
MMP2
IGF1
SCX

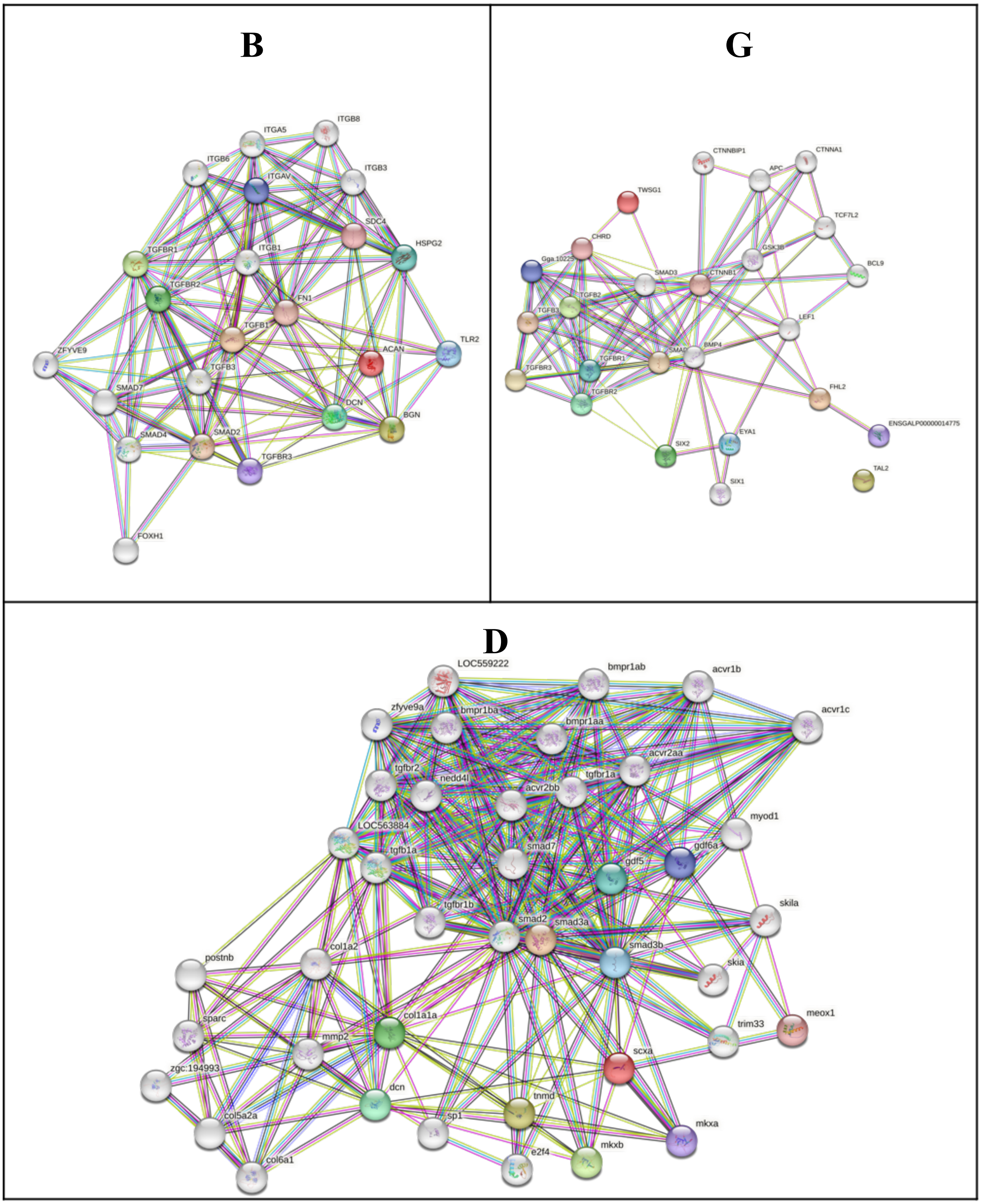
B
ITGB8
ITGA5
ITGB6
ITGAV
ITGB3
SDC4
HSPG2
TGFBR1
ITGB1
TGFBR2
FN1
TGFB1
ACAN
TLR2
ZFYVE9
TGFB3
SMAD7
DCN
BGN
SMAD4
SMAD2
TGFBR3
FOXH1
G
CTNNBIP1
CTNNA1
APC
TWSG1
TCF7L2
CHRD
GSK3B
BCL9
SMAD3
CTNNB1
TGFB2
TGFB3
LEF1
BMP4
TGFBR1
TGFBR3
TGFBR2
FHL2
ENSGALP00000014775
EYA1
SIX2
TAL2
SIX1
D
LOC559222
bmpr1ab
acvr1b
acvr1c
zfyve9a
bmpr1ba
bmpr1aa
acvr2aa
tgfbr2
nedd4l
tgfbr1a
acvr2bb
myod1
LOC563884
smad7
gdf6a
tgfb1a
gdf5
tgfbr1b
skila
smad2
smad3a
smad3b
postnb
col1a2
skia
sparc
col1a1a
trim33
meox1
mmp2
scxa
zgc:194993
dcn
tnmd
col5a2a
sp1
mkxa
mkxb
e2f4
col6a1

**Supplementary File 7. Graphic depiction of the StringNETs.** Known and predicted protein–protein interactions related to the molecules of the enriched networks. The interactions include direct (physical) and indirect (functional) associations; they stem from computational prediction, from knowledge transfer between organisms, and from interactions aggregated from other (primary) databases. Specific abbreviations were used for each model organism. Mus musculus (m), Rattus norvegicus (r), Homo sapiens (h), Equus caballus (e), and Bos taurus (b) Gallus gallus (g) and Danio rerio (d).
