# Supplementary material for: Mammal comparative tendon biology: advances in regulatory mechanisms through a computational modeling
Source: Front Vet Sci. 2023 Apr 27;10:1175346. doi: 10.3389/fvets.2023.1175346 (PMC10174257; doi:10.3389/fvets.2023.1175346)
Supplement: Supplementary file 8 [file Data_Sheet_8.pdf]

Supplementary File 8

Mammal comparative tendon biology: advances in regulatory mechanisms through a computational modelling

Alessia Peserico[1#*], Barbara Barboni[1#], Valentina Russo[1], Nicola Bernabò[1], Mohammad El Kathib[1], Giuseppe Prencipe[1], Adrián Cerveró-Varona[1], Arlette Alina Haidar Montes[1], Melisa Faydaver[1], Maria Rita Citeroni[1], Paolo Berardinelli[1], Annunziata Mauro[1].

[1]Unit of Basic and Applied Sciences, Department of Bioscience and Technology for Food, Agriculture and Environment, University Teramo, Via R. Balzarini 1, 64100 Teramo, Italy

# These authors equally contributed to this work

*** Correspondence:**
Alessia Peserico
apeserico@unite.it

Mus musculus Enriched TendonNET

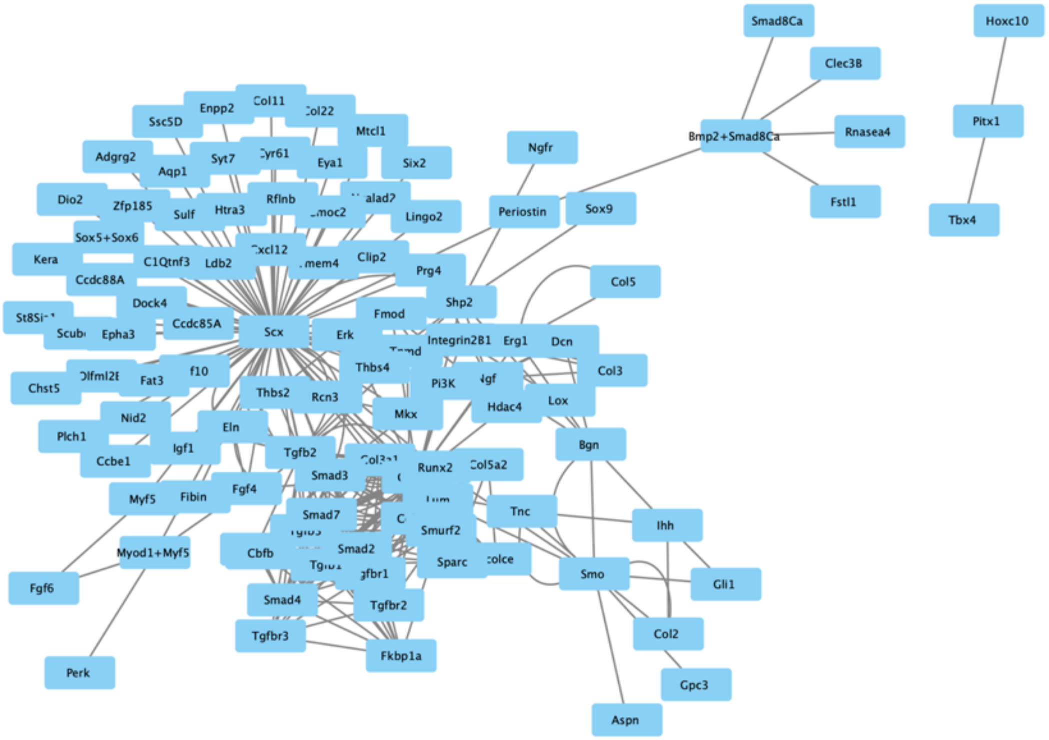

Rattus norvegicus Enriched TendonNET

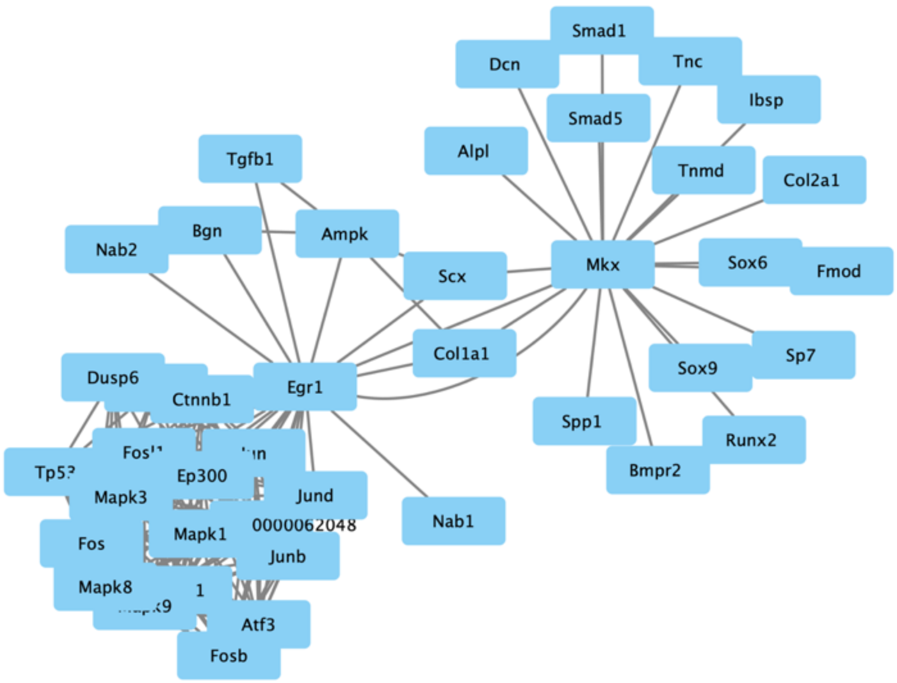

Homo sapiens Enriched TendonNET

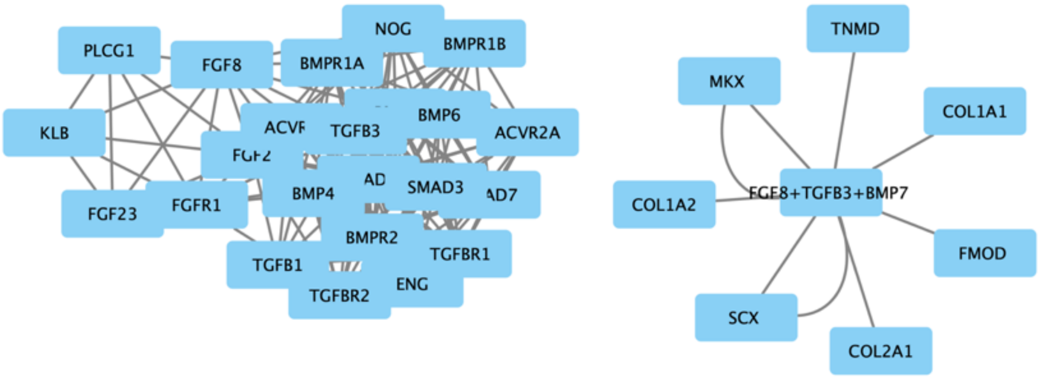

Equus caballus Enriched TendonNET

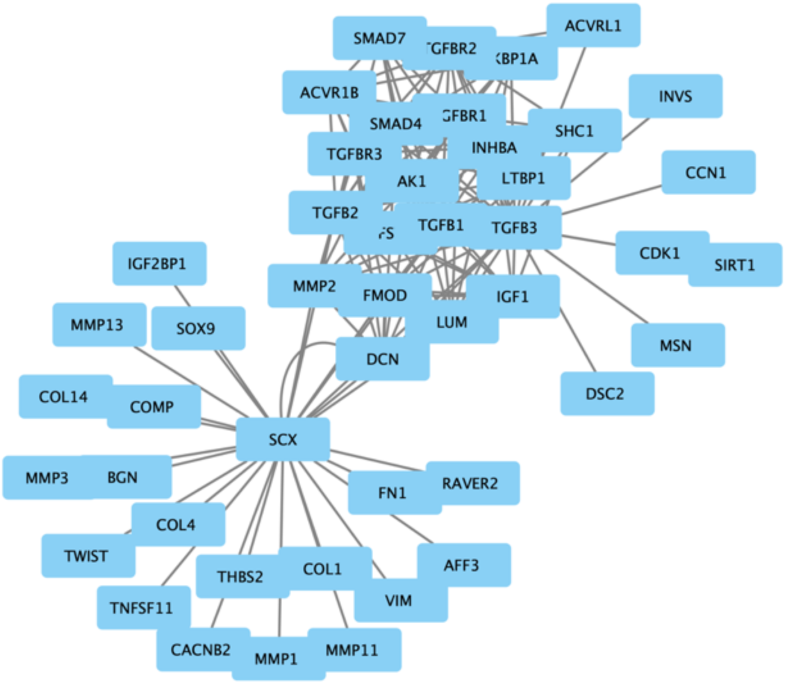

Bos taurus Enriched TendonNET

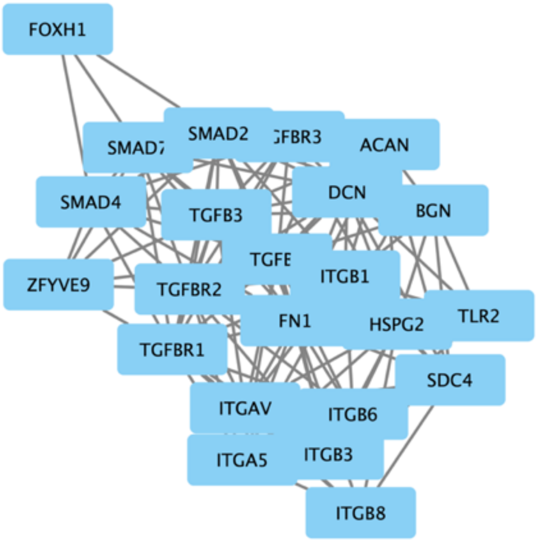

Gallus gallus Enriched TendonNET

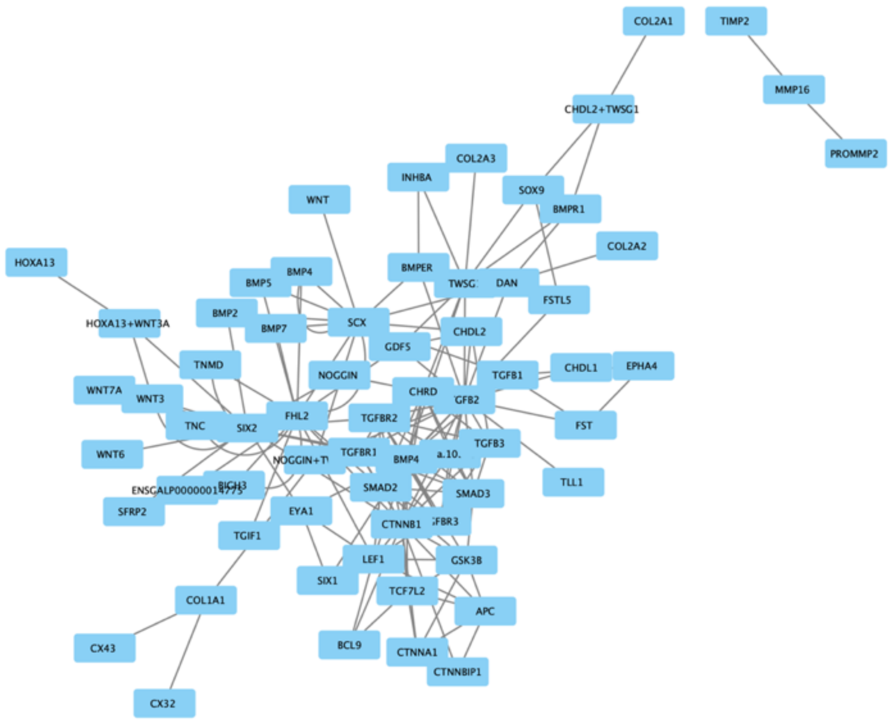

Danio rerio Enriched TendonNET

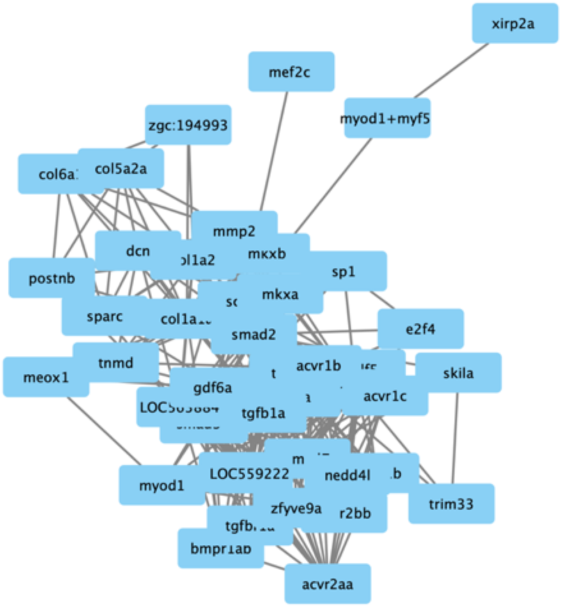

Supplementary File 8. Graphic depiction of the *Enriched* TendonNETs. Cytoscape network images were reported for each specie specific *Enriched* TendonNETs.
